# Tuberculosis treatment discontinuation and symptom persistence: an observational study of Bihar, India’s public care system covering >100,000,000 inhabitants

**DOI:** 10.1186/1471-2458-14-418

**Published:** 2014-05-01

**Authors:** Kimberly S Babiarz, Sze-chuan Suen, Jeremy D Goldhaber-Fiebert

**Affiliations:** 1 Centers for Health Policy and Primary Care and Outcomes Research, Stanford University, Stanford, CA, USA; 2 Department of Management Science and Engineering, Stanford University, Stanford, CA, USA

**Keywords:** Tuberculosis, Treatment discontinuation, Treatment default, Symptom persistence, Treatment duration, India

## Abstract

**Background:**

The effectiveness of India’s TB control programs depend critically on patients completing appropriate treatment. Discontinuing treatment prior to completion can leave patients infectious and symptomatic. Developing strategies to reduce early discontinuation requires characterizing its patterns and their link to symptom persistence.

**Methods:**

The 2011 BEST-TB survey (360 clusters, 11 districts) sampled patients (n = 1007) from Bihar’s public healthcare system who had initiated treatment >6 months prior to being interviewed, administering questionnaires to patients about TB treatment duration and symptoms, prior treatment, and sociodemographic characteristics. Multivariate logistic regression models estimated the risk of treatment discontinuation for these characteristics. Similar models estimated probabilities of symptom persistence to 25 weeks post-treatment initiation adjusting for the same predictors and treatment duration. All models included district fixed effects, robust standard errors, and adjustments for the survey sampling design. Treatment default timing and symptom persistence relied solely on self-report.

**Results:**

24% of patients discontinued treatment prior to 25 weeks. Higher likelihood of discontinuation occurred in those who had failed to complete previous TB treatment episodes (aOR: 4.77 [95% CI: 1.98 – 11.53]) and those seeing multiple providers (3.67 per provider [1.94 – 6.95]). Symptoms persisted in 42% of patients discontinuing treatment within 5 weeks versus 28% for completing 25 weeks of treatment. Symptom persistence was more likely for those with prior TB treatment (aOR: 5.05 [1.90 – 13.38]); poorer patients (2.94 [1.51 – 5.72]); and women (1.79 [1.07 – 2.99]). Predictors for treatment discontinuation prior to 16 weeks were similar.

**Conclusions:**

Premature TB treatment discontinuation and symptom persistence is particularly high among individuals who have failed to complete treatment for a prior episode. Strategies to identify and promote treatment completion in this group appear promising. Likewise, effective TB regimens of shortened duration currently in trials may eventually help to achieve higher treatment completion rates.

## Background

Tuberculosis (TB) represents a major global health burden of particular concern given its growing resistance to available drugs. India bears the world’s largest TB burden with 3,000,000 cases in 2009 [1]. India’s substantial efforts to address TB through its public healthcare system have led to declines in TB prevalence and incidence [2]. More recently, India’s public healthcare system has also increased its efforts to address the emergence of multidrug resistant (MDR) TB (MDR is defined as TB resistant to at least rifampicin and isoniazid) [1,3-7].

Premature TB treatment discontinuation (i.e., default) hampers TB control efforts. Early discontinuation implies exposure to TB drugs for durations insufficient to effect cure. Such exposures may also select for drug resistant strains [8,9] that require treatment that is longer and more toxic than for non-MDR TB [9-14].

Because TB treatment is lengthy, complicated, and potentially toxic, premature treatment discontinuation among a fraction of patients is almost inevitable, prompting efforts to reduce the current length of treatment, which exceeds 25 weeks [2]. Development of shorter treatments (e.g., 4-month regimens containing quinolones) [15,16] could potentially reduce the chance of premature discontinuation and drug resistance. However, success depends partly on the timing of discontinuation – if discontinuation happens frequently in the first month of treatment, shortening regimens to even 2 months would not change cure rates. The patterns and timing of treatment discontinuation and the factors predicting discontinuation are not well characterized in many high TB burden settings.

In India, treatment discontinuation is high as patients with active TB disease are often poor, socially disadvantaged, and under-educated; alcohol use also contributes to higher discontinuation rates [17-24]. Many patients first receive ineffective care in the private health sector, which saps resources and delays their entry into the public system [25-27]. As their symptoms improve with effective treatment, continued costs, inconvenience, and toxicity risks may make remaining on treatment increasingly unappealing. For these reasons, treatment discontinuation likely differs among those who have been treated previously in the private sector and is likely linked to the pace at which patients’ symptoms improve. Understanding differences in patterns of discontinuation for treatment-experienced and for treatment-naïve patients is needed because multiple treatment exposures increase the probability of accumulating mutations resistant to specific medications, raising the risk of MDR TB.

Bihar exemplifies the challenges of India’s TB epidemic given its historically high levels of poverty and poor health outcomes [28,29]. Bihar’s population exceeds 100 million people [30] (approximately 10% of India’s total), its public healthcare system registered 76,484 patients for TB treatment in 2011 [30], and it likely sees an appreciable proportion of drug resistant TB [30-33].

The present study focuses on patients treated for TB in Bihar’s public healthcare system. It had two goals: 1) characterize treatment discontinuation and symptom persistence despite treatment; and 2) identify characteristics of TB patients and their healthcare experiences that predict these outcomes and are potentially amenable to interventions to reduce rates of premature treatment discontinuation.

## Methods

### Ethics statement

The study analyzes data from the Bihar Evaluation of Social Franchising and Telemedicine - Tuberculosis (BEST-TB) survey which received Institutional Review Board review and approvals from Duke University and Stanford University in the U.S. and from Society for the Promotion of Ethical Clinical Trials – Ethics Review Board (SPECT-ERB) in India. Research permissions were also granted by the Governments of India and of the State of Bihar. All subjects provided written informed consent.

### Survey, sample and data collection

**Figure 1:**
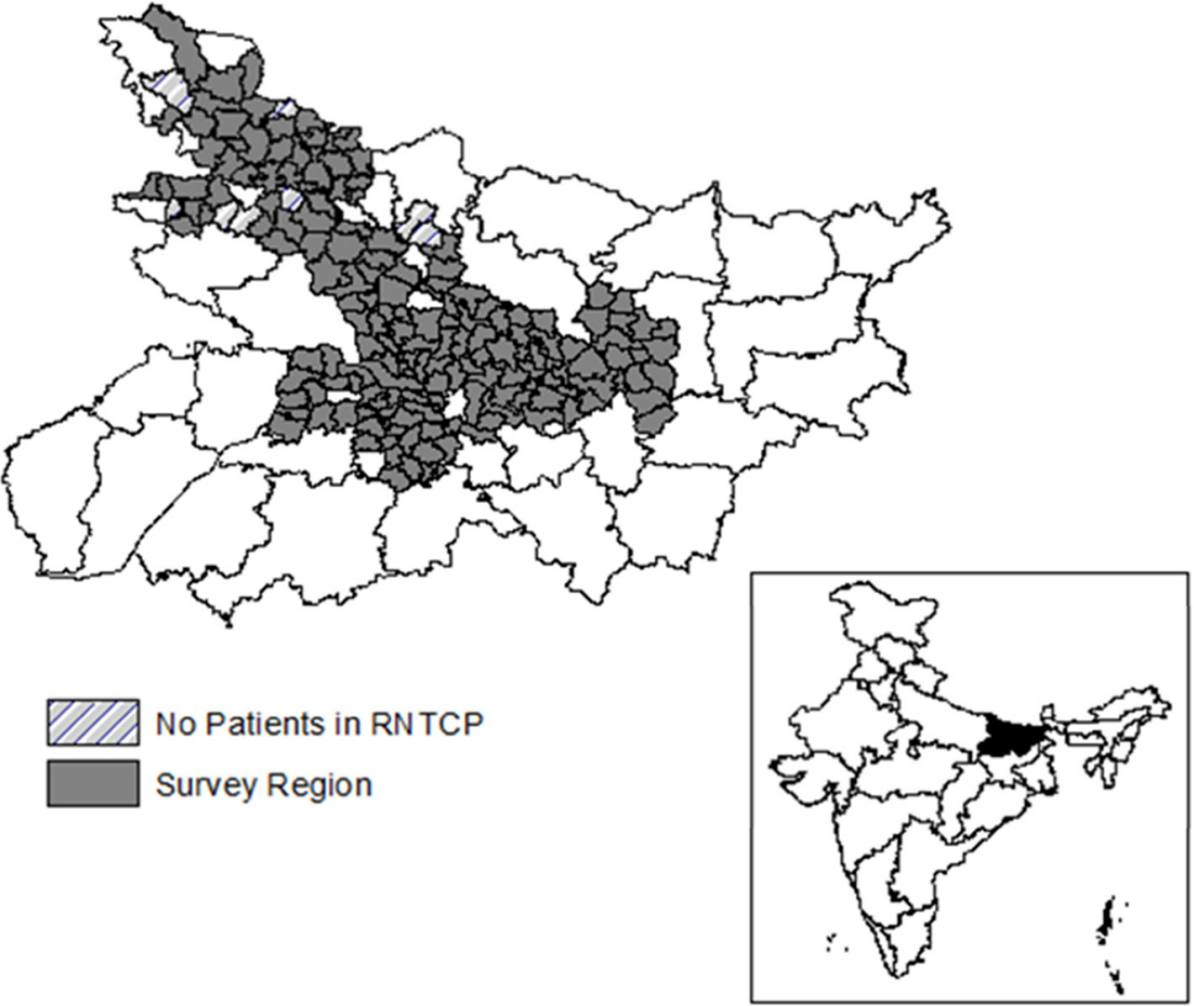
**Map of regions surveyed within districts of Bihar (Inset map showing location of Bihar within India).** The figure shows the map of Bihar and its districts. Gray areas represent the survey regions containing the 360 study clusters. Hashed areas represent survey regions with no TB patients eligible for study inclusion. The inset map of India shows the state of Bihar (Black) along with all other Indian states.

The 2011 BEST-TB survey, a cross-sectional survey of TB patients treated in the public healthcare system in 11 districts of Bihar, India, provided data for the present analysis. The survey occurred in 360 rural clusters (Figure 1) whose sampling was determined as part of the baseline for a larger evaluation of interventions for a variety of diseases [34]. To represent patients receiving care in the public TB healthcare system, we constructed the sampling frame using TB patient lists from all Primary Health Centers (PHCs) within the study clusters as India’s Revised National Tuberculosis Control Programme (RNTCP) provides standard TB medications and care free of charge according to the Directly Observed Treatment Short Course (DOTS) protocol to an estimated 60% of TB cases [35-39]. For potential inclusion in the study, enumerators recorded names and addresses of all TB patients entered on PHC lists within the past year (July 2010- July 2011) living within study clusters (i.e., village catchments representing part of the catchment served by a given PHC). Combining these names for each cluster generated the cluster’s sample list. From each cluster’s list, 6 patients were randomly sampled for study inclusion; all patients were included from cluster lists with fewer than 6 names. While the planned sample size was 2160, a review of all PHC records indicated that 20 clusters had no patients treated in the previous year and living within a study catchment area, and 22% of cluster lists had ≤6 names. Hence, 1691 patients were selected for subsequent household survey. There were no additional exclusion criteria.

Response rates to enumerators visiting and surveying each patient’s household were high. Just 44 households (2.6%) were not surveyed because they could not be located or no competent respondent was found. Of the remaining 1647 households, 5 refused (0.3%) and 68 reported that the TB patient had died (4.0%). Additionally, 28 patients provided incomplete data and were excluded. Household surveys were completed for 1546 patients (91.4% response rate of households contacted for inclusion). Because the present analyses focus on the treatment experience of patients in the RNTCP public system which according to DOTS treatment category should be at least 25 weeks in length, we restricted our analyses to patients initiating treatment at least 6 months prior to the interview (1007 patients).

Based on each TB patient’s responses to the questionnaire, we reconstructed a timeline of his or her illness and treatment relying solely on patient self-report. Because of the high variability of data recorded in the treatment records at the PHCs in our prior piloting of the survey, we did not use these records for our analysis. Instead, we relied solely on patients reporting their detailed experiences of common TB symptoms: persistent cough (≥3 weeks), coughing blood, fever, weight loss, night sweats, chronic fatigue, and loss of appetite [40,41]. We recorded their reported number of days since the onset of each symptom and the number of days each persisted. We recorded the number of days each patient waited to seek care, before a diagnosis was received, and before beginning treatment. Finally, we recorded the number of days each patient reported remaining on treatment in the public system. We refer to the patient’s most recent episode of TB as the *current episode*, regardless of whether the patient remained ill at the time of survey. Patients also provided information about treatment for TB prior to the current episode, demographic characteristics, household composition and household assets.

### Outcomes

Main outcomes were treatment discontinuation prior to 25 weeks (hereafter simply “treatment discontinuation”) and symptom persistence. Given policy interest in shorter treatment regimens, we repeated our analysis for treatment discontinuation prior to 16 weeks. Given policy interest in shorter treatment regimens and heterogeneity in current treatment regimens, we repeated our analysis for treatment discontinuation prior to 16 weeks for the entire sample and prior to 32 weeks for those with prior TB treatment episodes. Symptom persistence was defined as having ≥1 TB symptom 25 weeks after treatment initiation. We also explored an alternate model using the number of TB symptoms present at 25 weeks.

### Predictors

Main predictors of treatment discontinuation and symptom persistence were reporting treatment for a prior episode of TB and completion of prior TB treatment. It is unclear whether those with prior TB treatment are more or less likely to complete treatment for their current episode. If patients are already familiar with treatment, those choosing to repeat treatment may remain in care longer than first-time patients. However, patients who have undergone prior TB treatment may experience treatment fatigue and discontinue earlier. Failure to complete the prior TB treatment is hypothesized to predict early discontinuation from the current treatment episode. Because repeated treatment can select for MDR particularly if treatment is not completed and MDR is more difficult to cure [32,42], we also hypothesize that both prior TB treatment and failing to complete prior treatment predict symptom persistence at the end of the current episode of treatment, while noting that there are other reasons for symptoms to persist (residual lesions even after cure; other respiratory ailments) which we cannot distinguish.

#### ***TB illness, treatment, and costs***

A patient’s total number of symptoms at the time of seeking care proxied for illness severity at baseline. We controlled for treatment delays in two ways [43-51]. First, we used the number of weeks between symptom onset and treatment initiation. Second, we used the number of treatment providers seen for the current episode. Additionally, although treatment for TB through RNTCP should be free to the patient, we included indicators for whether the patient reported paying for service or medication fees, for travel to the site of care, or both [37,52]. We hypothesized that more severely ill patients remain in care longer, but are also more likely to have symptoms persist. The relationship of treatment discontinuation to both delays in seeking treatment and for having seen additional providers prior to treatment in the public system was unclear. If illness severity worsens with longer delays, patients may remain in care longer. However, treatment delays may indicate unobserved characteristics that imply higher discontinuation rates. We hypothesize that both treatment delays and seeing more providers are associated with greater symptom persistence. We hypothesize that paying for service and medications fees and travel costs are associated with higher treatment default and consequently with greater symptom persistence.

#### ***Patient and household characteristics***

We included patient sex, religion (Hindu vs. non-Hindu), social group (scheduled caste, scheduled tribe, or other backwards class vs. other castes), age (years), and education (0 if illiterate and number of years of education among the literate). We hypothesize that men, Hindus, those with higher education and those belonging to “other castes” remain in care longer and are less likely to have symptom persistence [37,53-55]. Household assets proxied for wealth based on a wealth index constructed using a standard principle components analysis of assets [56] and then grouped households into relative wealth tertiles, acknowledging that most TB households are not wealthy compared to India’s general population [57]. We accounted for household composition in two ways: the number of people residing in the home and number of children below age 5 in the home. We hypothesized that patients from the poorest households are more likely to discontinue treatment prior to 25 weeks [37,53-55,58]. If larger households have more resources to compensate for lost work and time devoted to TB treatment, then patients from larger households will remain in care longer. However, the demands associated with children in the household imply higher opportunity costs of continued treatment. We therefore expect that number of children is positively correlated with treatment discontinuation. The combined effects of household composition on symptom persistence were unclear.

### Statistical analysis

We predicted the likelihood of ending treatment before 25 weeks using a multivariate logistic regression model. We estimated the model using all 1007 patients controlling for prior TB treatment and treatment completion, repeating the analysis separately for 196 patients with prior TB treatment and for 811 with no prior treatment to examine differential effects of other predictors within these subgroups. The Supporting Information also reports a linear probability model of treatment discontinuation prior to 25 weeks and both logistic regression and linear probability models of treatment discontinuation prior to 16 weeks of treatment for the entire sample and prior to 32 weeks for those with prior TB treatment episodes. We likewise assessed determinants of symptom persistence 25 weeks after treatment initiation using a multivariate logistic model. The Supporting Information also reports a negative binomial model for the number of symptoms persisting 25 weeks post treatment initiation. For symptom persistence, we included the same predictors as for default along with duration in treatment covariates. We explored multiple definitions of duration of treatment including an indicator for completing ≥180 days of treatment; number of weeks of completed treatment; and indicators for completing 0-8 weeks, 9-16 weeks, and >16 weeks.

For all analyses, the multivariate models did not employ variable selection techniques but instead included all predictors for which there was a theory-predicted relationship to the outcome. Likewise, for all analyses, we included district-level fixed effects to control for unobserved characteristics of healthcare systems, geographic areas, and patient populations within each district. We also adjusted for survey design using inverse probability sampling weights and calculated robust standard errors clustered by sampling unit [59].

## Results

Table 1 reports descriptive statistics for the sample of 1007 TB patients receiving treatment in the public healthcare system in Bihar who initiated treatment >180 days prior to their household interview. Their characteristics were generally consistent with those reported for other Indian TB populations [35-39].

**Table 1:** Description of TB patients

		**Overall**	**First time**	**Repeat**	**Repeat**
			**TB patients**	**TB patients: completed prior treatment**	**TB patients: incomplete prior treatment**
**N**		1007	811	157	39
**Percent of all patients**			80%	16%	4%
**Patient characteristics**					
**Male**	%	67.4%	68.3%	66.3%	54.9%
**Mean age**	Yrs	37.4	37.4	37.2	36.6
**Literate**	%	36.9%	39.1%	27.3%	31.8%
**Education: mean amount of schooling (If Literate)**	Yrs	7.15	7.20	6.66	7.79
**Scheduled Caste, scheduled Tribe, other backward class**	%	91.5%	91.3%	92.3%	90.3%
**Hindu**	%	88.9%	89.3%	88.2%	84.2%
**Household characteristics**					
**Household size**	#	6.59	6.43	7.36	6.37
**Number of children under 5**	#	1.64	1.59	1.87	1.70
**Treatment and illness**					
**Total weeks from symptom onset to treatment initiation**	Wks	6.05	6.01	6.33	5.63
**Number of providers visited**	#	1.07	1.04	1.18	1.25
**Any treatment or medication fees**	%	63.9%	59.2%	85.3%	68.2%
**Any travel costs**	%	79.4%	79.5%	78.4%	82.0%
**Mean total cost of treatment among those paying any costs/fees**	Rupees	318	296	385	456
**2 or fewer symptoms at treatment initiation**	%	46.7%	50.5%	23.1%	69.9%
**3-4 symptoms at treatment initiation**	%	18.7%	18.3%	21.6%	14.1%
**≥5 symptoms at treatment initiation**	%	34.6%	31.1%	55.4%	16.0%

### Treatment discontinuation

**Figure 2:**
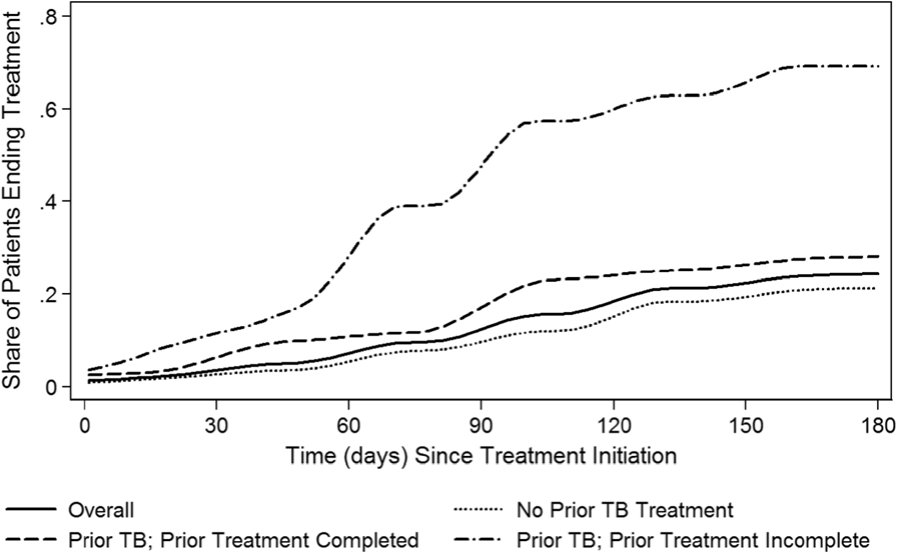
**Cumulative risk of treatment default stratified by prior TB treatment and prior treatment completion.** The figure shows the proportion of TB patients who have ended treatment during the first 25 weeks of treatment. In addition to showing the overall rate of treatment default (solid line), separate curves are shown for patients with no prior TB treatment episodes, patients with prior TB episodes where they completed prior treatment, and patients with prior TB episodes where they did not complete treatment.

Individuals who did not complete treatment for prior episodes of TB have differentially higher probabilities of discontinuing treatment prior to 25 weeks in their current TB treatment episode compared to those with no prior episodes and those who completed treatment for their prior episodes (Figure 2). More than 69% [95% CI 50-89%] of the 39 patients who reported that they had not completed their previous TB treatment also failed to complete at least 25 weeks of their current treatment (median time to default 17 weeks) compared to 28% [95% CI: 16-40%] of the 157 patients who reported completing their previous treatment and 21% [95% CI: 17-26%] of the 811 patients treated for the first time (median time to default 25 weeks and 24 weeks respectively).

Individuals who had prior TB episodes but did not complete treatment have higher probability of discontinuing their current treatment episodes after adjusting for multiple factors (adjusted Odds Ratio (aOR): 4.77 [95% CI: 1.98 – 11.53]) (Table 2). Discontinuation probabilities of those who completed prior TB treatment are similar to those without prior treatment (approximately 1.05 after combining the aOR for prior TB with the interaction aOR for completing prior treatment: 0.22 [95% CI: 0.08 – 0.59]). Seeing more treatment providers for the current episode of TB predicts higher probabilities of treatment discontinuation (aOR: 3.67 per provider seen [95% CI: 1.94 – 6.95]). When one considers the effects of paying for treatment costs, travel costs, and the amount paid, paying anything predicts higher rates of treatment discontinuation, while those who are able to pay more and choose to do so are less likely to discontinue treatment.

**Table 2:** Likelihood of treatment discontinuation <25 weeks after treatment initiation: logistic regression results

	**Univariate regression**		**Multivariate regressions**					
	**All patients**		**All patients**		**Patients with prior TB**		**Patients with no prior TB**	
	**OR**	**(95% CI)**	**OR**	**(95% CI)**	**OR**	**(95% CI)**	**OR**	**(95% CI)**
**Prior TB status**								
**Prior TB treatment episode**	6.15*	(2.60 - 14.53)	4.77*	(1.98 - 11.53)				
**Prior TB & completed prior treatment**	0.23*	(0.09 - 0.60)	0.22*	(0.08 - 0.59)	0.15*	(0.03 - 0.73)		
**Current illness treatment and illness characteristics**								
**Total weeks from symptom onset to treatment initiation**	1.00	(0.95 - 1.05)	1.02	(0.97 - 1.07)	1.04	(0.90 - 1.20)	1.02	(0.97 - 1.08)
**Number of Providers visited**	4.68*	(2.64 - 8.27)	3.67*	(1.94 - 6.95)	3.08	(0.46 - 20.45)	5.62*	(2.32 - 13.66)
**Treatment or medication fees**	6.83*	(2.18 - 21.41)	4.60*	(1.38 - 15.40)	1.13	(0.08 - 16.48)	6.32*	(1.43 - 28.00)
**Travel costs**	2.70*	(1.07 - 6.82)	2.55*	(1.03 - 6.33)	1.43	(0.13 - 16.08)	4.39*	(1.36 - 14.13)
**Treatment, medication and travel costs**	0.21*	(0.06 - 0.69)	0.34	(0.10 - 1.18)	1.04	(0.05 - 21.04)	0.19	(0.03 - 1.06)
2 or fewer symptoms at treatment initiation**	1.18	(0.75 - 1.85)	1.62	(0.96 - 2.73)	4.20*	(1.26 - 13.92)	1.29	(0.76 - 2.21)
3-4 symptoms at treatment initiation**	1.38	(0.74 - 2.56)	1.54	(0.83 - 2.86)	2.55	(0.66 - 9.95)	1.16	(0.65 - 2.09)
**Patient and household characteristics**								
**Male**	1.29	(0.85 - 1.95)	1.32	(0.82 - 2.14)	1.02	(0.39 - 2.69)	1.34	(0.81 - 2.22)
**Age**	0.96	(0.91 - 1.00)	0.96	(0.91 - 1.01)	0.84	(0.70 - 1.01)	0.98	(0.93 - 1.03)
**Age squared**	1.00	(1.00 - 1.00)	1.00	(1.00 - 1.00)	1.00*	(1.00 - 1.00)	1.00	(1.00 - 1.00)
**Education**	1.01	(0.96 - 1.06)	0.99	(0.93 - 1.06)	0.75*	(0.58 - 0.98)	1.01	(0.95 - 1.07)
**Hindu**	0.83	(0.51 - 1.35)	0.81	(0.47 - 1.40)	0.25*	(0.08 - 0.78)	0.97	(0.51 - 1.84)
**Scheduled Caste, Tribe, other backwards class**	0.87	(0.53 - 1.44)	0.84	(0.47 - 1.50)	0.36	(0.08 - 1.71)	1.02	(0.51 - 2.02)
**Number of Kids**	0.95	(0.82 - 1.09)	1.02	(0.86 - 1.21)	0.80	(0.53 - 1.20)	1.08	(0.89 - 1.31)
**Household size**	0.97	(0.88 - 1.06)	0.96	(0.86 - 1.07)	1.35	(0.97 - 1.88)	0.92	(0.81 - 1.04)
**Poor**	1.09	(0.68 - 1.74)	0.97	(0.56 - 1.66)	0.76	(0.14 - 4.11)	1.27	(0.73 - 2.19)
**Middle income**	0.70	(0.36 - 1.36)	0.86	(0.48 - 1.54)	0.99	(0.30 - 3.20)	0.95	(0.56 - 1.61)
**Observations**	1007		1007		196		811	

*p < 0.05.

**Comparator group is ≥5 Symptoms at Treatment Initiation.

Predictors of treatment discontinuation are distinct when the analysis is repeated separately for those with and without prior TB treatment. Significant predictors of higher probability of treatment discontinuation among those treated for TB previously are having not completed the prior episode of TB treatment along with personal characteristics including having fewer TB symptoms at the time of treatment initiation, younger age, less education, and being non-Hindu. In contrast, for those who had not been treated previously, significant predictors of greater probability of treatment discontinuation were not individual characteristics including number of symptoms as a marker for illness severity but rather care seeking pathways like the number of providers seen for the current episode of TB treatment and payment for treatment and related costs. The direction of these relationships generally remains consistent when analyses are repeated using a linear probability model though at times significance attenuates (see Additional file 1).

The predictors of treatment discontinuation within 4 months of starting treatment are consistent with the results of the main analysis – having had prior TB treatment that was not completed (aOR: 4.63 [95% CI: 1.88 – 11.42]), and seeing more providers (aOR per provider: 5.60 [95% CI: 2.18 – 14.37]) (see Additional files 2 and 3).

### Symptom persistence

**Figure 3:**
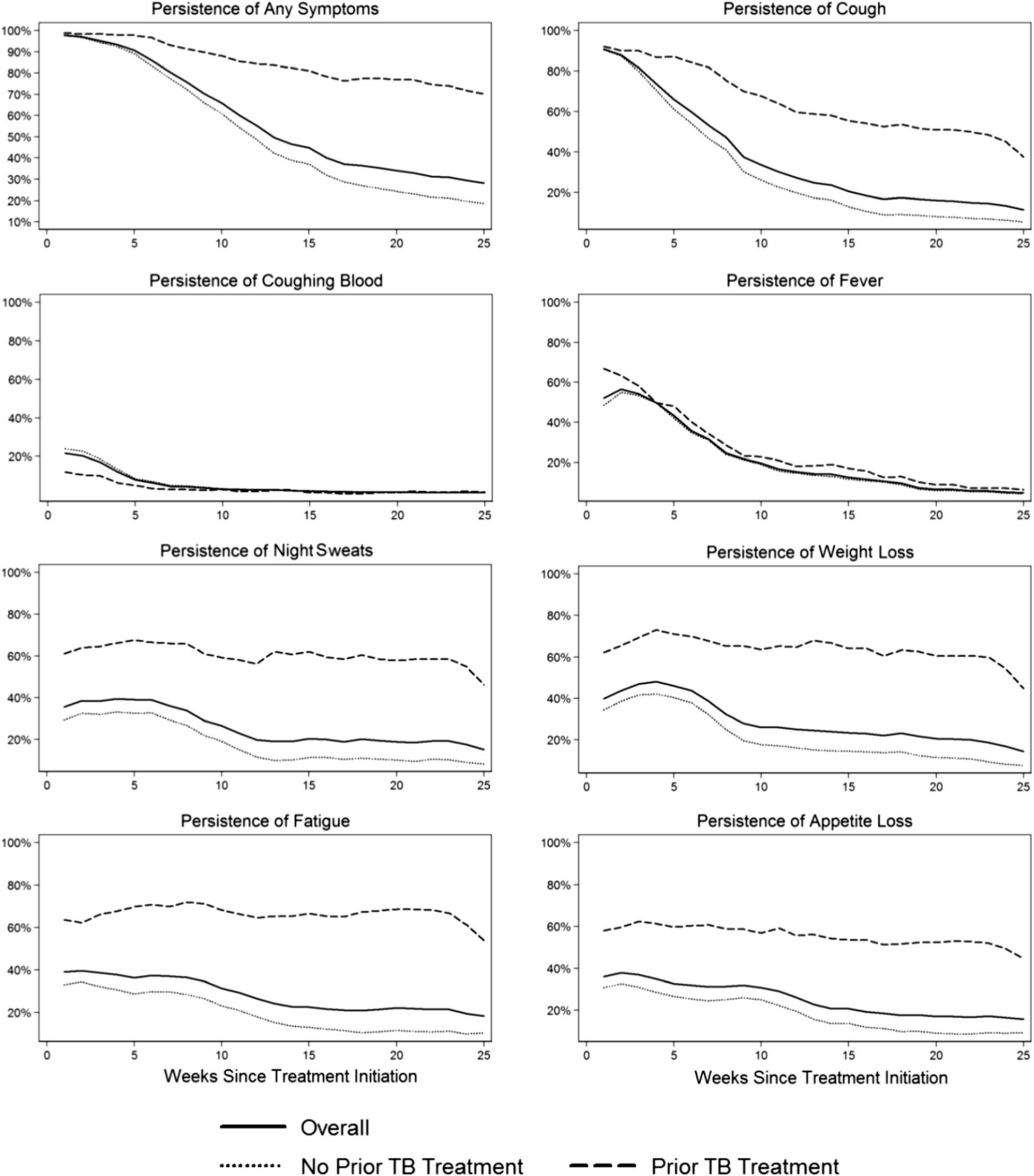
**Proportion of TB patients with symptoms persisting by time since treatment initiation.** The figure shows the proportion of patients with any symptoms and with specific symptoms a given number of weeks after treatment initiation for all patients and stratified by patients with and without prior TB episodes.

Symptoms persisted 25 weeks after treatment initiation for 28% of patients despite remaining in treatment for at least 25 weeks (Figure 3). Among patients discontinuing treatment early, symptoms persisted in 42% of patients completing ≤5 weeks, 33% of patients completing ≤10 weeks, and 30% of patients completing ≤18 weeks. Symptoms most likely to persist despite 25 weeks of treatment include fatigue (18% of patients), appetite loss (16%), night sweats (15%), weight loss (14%) and coughing (11%) (Figure 3). Persistence of symptoms followed different trajectories for those with and without prior TB treatment with symptoms more likely to persist for those with prior TB treatment (Figure 3).

TB symptom persistence 25 weeks post treatment initiation is more likely for those with prior TB treatment (aOR: 5.05 [95% CI: 1.90 – 13.38]), poorer patients (aOR for lowest wealth tertile among TB households: 2.94 [95% CI: 1.51 – 5.72]), and women (aOR of men compared to women: 0.56 [95% CI: 0.33 – 0.93]) (Table 3). For those with prior TB, the main predictors of symptom persistence are the number of treatment providers seen (aOR per provider seen: 3.76 [95% CI: 1.00 – 14.11]) (Table 3). For those with no prior TB, the main predictors of symptom persistence are not actually paying for treatments, medications and travel which is likely a marker of poverty in terms of the inability to pay, and poverty as explicitly measured by being in the lowest wealth tertile among TB households (aOR: 2.91 [95% CI: 1.50 – 5.68]) (Table 3). Notably, number of symptoms at treatment initiation did not predict symptom persistence.

**Table 3:** Likelihood of symptom persistence (symptoms at 25 weeks after treatment initiation): logistic regression results

	**Univariate regression**		**Multivariate regression**					
	**All patients**		**All patients**		**Patients with prior TB**		**Patients with no prior TB**	
	**OR**	**(95% CI)**	**OR**	**(95% CI)**	**OR**	**(95% CI)**	**OR**	**(95% CI)**
**Prior TB status**								
**Prior TB treatment episode**	4.59*	(2.17 - 9.70)	5.05*	(1.90 - 13.38)				
**Prior TB & completed prior treatment**	1.63	(0.58 - 4.62)	2.40	(0.78 - 7.37)	0.81	(0.26 - 2.48)		
**Current illness treatment and illness characteristics**								
**Total weeks from symptom onset to treatment initiation**	1.01	(0.97 - 1.05)	0.96	(0.91 - 1.01)	0.95	(0.86 - 1.05)	0.96	(0.89 - 1.02)
**Number of providers visited**	2.73*	(1.42 - 5.25)	2.06	(0.93 - 4.56)	3.76*	(1.00 - 14.11)	1.10	(0.25 - 4.86)
**Treatment or medication fees**	2.79*	(1.16 - 6.71)	1.17	(0.42 - 3.25)	1.30	(0.12 - 14.46)	1.59	(0.43 - 5.92)
**Travel costs**	2.16*	(1.10 - 4.27)	1.89	(0.91 - 3.92)	0.73	(0.06 - 8.66)	1.74	(0.81 - 3.70)
**Treatment, medication and travel costs**	0.13*	(0.05 - 0.33)	0.16*	(0.06 - 0.46)	1.30	(0.08 - 22.00)	0.10*	(0.03 - 0.39)
**Remained in care 0-8 weeks**	1.59	(0.73 - 3.43)	0.50	(0.19 - 1.35)	0.35	(0.08 - 1.55)	0.70	(0.17 - 2.89)
**Remained in care 9-16 weeks**	0.90	(0.31 - 2.61)	0.90	(0.29 - 2.76)	0.29	(0.07 - 1.17)	2.12	(0.75 - 6.02)
2 or Fewer symptoms at treatment initiation**	0.95	(0.57 - 1.58)	1.10	(0.64 - 1.89)	0.89	(0.28 - 2.84)	1.93	(0.96 - 3.88)
3-4 symptoms at treatment initiation**	0.57	(0.28 - 1.16)	0.50	(0.18 - 1.43)	0.42	(0.12 - 1.45)	1.11	(0.37 - 3.35)
**Patient and household characteristics**								
**Male**	0.66*	(0.45 - 0.96)	0.56*	(0.33 - 0.93)	0.41	(0.15 - 1.12)	0.76	(0.45 - 1.30)
**Age**	0.97	(0.92 - 1.01)	0.97	(0.91 - 1.03)	0.97	(0.88 - 1.07)	0.96	(0.90 - 1.03)
**Age squared**	1.00*	(1.00 - 1.00)	1.00	(1.00 - 1.00)	1.00	(1.00 - 1.00)	1.00	(1.00 - 1.00)
**Education**	0.95	(0.91 - 1.00)	1.02	(0.95 - 1.09)	0.99	(0.87 - 1.13)	0.99	(0.92 - 1.06)
**Hindu**	0.65	(0.41 - 1.03)	0.54	(0.27 - 1.08)	0.61	(0.10 - 3.94)	0.49	(0.22 - 1.08)
**Scheduled Caste, Tribe, other backwards class**	0.85	(0.52 - 1.38)	0.90	(0.51 - 1.59)	0.71	(0.09 - 5.56)	0.92	(0.46 - 1.86)
**Number of Kids**	1.02	(0.88 - 1.19)	1.03	(0.84 - 1.28)	0.78	(0.54 - 1.12)	1.14	(0.88 - 1.49)
**Household size**	1.06	(0.96 - 1.17)	0.99	(0.87 - 1.13)	1.18	(0.89 - 1.57)	0.90	(0.77 - 1.06)
**Poor**	2.37*	(1.44 - 3.91)	2.94*	(1.51 - 5.72)	1.89	(0.66 - 5.38)	2.91*	(1.49 - 5.68)
**Middle income**	0.86	(0.45 - 1.63)	1.02	(0.52 - 2.00)	0.81	(0.24 - 2.73)	0.84	(0.48 - 1.47)
**Observations**	1007		1007		196		811	

*p < 0.05.

**Comparator group is ≥5 Symptoms at Treatment Initiation.

Results for predictors of symptom persistence remain largely consistent when the number of symptoms present 25 weeks after treatment initiation is used as the outcome, when different specifications for time in treatment are used as predictors in both linear probability models and logistic regression models, and when we estimate a model of symptom persistence 16 weeks after treatment initiation (see Additional files 4,5 and 6).

## Discussion

24% of surveyed TB patients reported failing to complete at least 25 weeks of treatment in Bihar’s public healthcare system. The rate of premature treatment discontinuation was nearly 59% for patients who had previously started but not finished TB treatment. High discontinuation rates are concerning given that a proportion of these patients that undergo inappropriately short treatment may remain infectious and because ineffective exposures to TB medications may select for drug resistant mutations. For 28% of patients, symptoms persist after 25 weeks of treatment; symptom persistence is even more common for those discontinuing treatment and for patients previously treated for TB, suggesting a lack of cure in some patients.

Our study contributes to the prior literature [17-24] and extends knowledge by connecting premature treatment discontinuation and symptom persistence. Main predictors of treatment discontinuation are consistent with prior studies as are those for symptom persistence. The link between treatment discontinuation and symptom persistence especially in patients receiving repeated TB treatment over multiple illness episodes is important because it illustrates a subgroup of patients who may benefit from further supportive interventions and who are important candidates for further TB and MDR TB control efforts.

TB treatment in the private healthcare sector plays a role in our analysis as a predictor of treatment discontinuation and symptom persistence but is also additionally important in its own right. Limited data from the private healthcare sector and from the studies evaluating public-private partnerships for TB treatment [60-62] suggest that, in these settings, treatment duration is often too short, provider switching too frequent, and delays in care too long. We know of no data examining symptom persistence after treatment in these settings. Given that approximately 40% of patients are treated in these settings even if they ultimately receive care in the public healthcare sector, studies similar to ours evaluating outcomes in the private healthcare sector in a representative patient population could provide substantial new insights to enable the improvement of TB care.

Our findings suggest areas for policy design. Strategies that increase cure rates and link private providers to the public system so patients receive prompt, effective treatment [47,60,63] could potentially reduce TB treatment discontinuation and symptom persistence. This is supported by our findings that in addition to higher treatment discontinuation rates and greater symptom persistence in those who have been treated for prior TB episodes, treatment discontinuation rates and symptom persistence for the current episode are higher among individuals who sought care with private providers before being treated in the public system. Likewise, programs that reach marginalized groups are needed given symptom persistence is higher among women and the poor.

Our findings regarding the timing of treatment discontinuation (Figure 2) also support the potential benefits of treatment regimens that achieve cure more rapidly and thus reduce required treatment duration [15,16]. Rates at which patients discontinue treatment are relatively constant over 25 weeks and do not appear to be especially high during the first few months. Further, predictors of treatment discontinuation before 16 weeks follow the same pattern as those for 25 weeks (Additional files 2 and 3). These findings suggest that reducing treatment duration from 25 weeks to 16 weeks could yield an absolute percentage point increase in treatment completion of almost 10%.

While one might expect highest default rates early on in treatment, at least for those individuals with prior TB treatment, the rate of default increases 45-90 days after treatment initiation (see Additional file 7). While one might have anticipated higher rates of discontinuation during the difficult period of injectable use, our finding could potentially be explained by patients believing that “real, necessary treatment” was over because they simply felt better or because they believed that injections constituted effective treatment and that phase of their treatment was over [64].

Our study has several limitations. Patients sampled are from Bihar only. While other states differ in terms of TB care and outcomes [2,30], Bihar’s population of 100 million represent roughly 10% of India’s total and is larger than most high burden countries. Patients are only sampled from the public healthcare sector, and, therefore, the study cannot comment on individuals who receive care only in the private sector, though other research shows high levels of provider switching in the private sector [39,46] and low levels of effective medication use [25-27]. Likewise, information on alcohol consumption in the BEST survey was limited and could not be included in the present analysis, though other prior studies have found that alcoholism is linked to TB treatment default [65,66].

An important study limitation is the reliance on self-report of default and default timing as well as TB status and symptoms without external validation. Our findings of default rates differ from those reported for comparable areas of Bihar and are generally substantially lower, though we note the appreciable heterogeneity across districts on numerous measures of program performance with some districts performing at 50% of other districts (Additional files 8,9,10 and 11) [67-72]. The difference in default rates could result from under-reporting of default in official government reports, over-reporting of default on the part of patients, or a combination of the two. Our study is unable to distinguish between these possibilities, though even if levels of default differ or are not perfectly representative of all of Bihar or other parts of India, differentially higher default rates among individuals who have had multiple exposures to TB drugs, as is also seen in official reports (Additional files 8,9,10 and 11), and ongoing symptoms are clearly of concern.

The study does not include clinical measures (i.e., sputum smears or drug sensitivity testing) or other administrative records that were found to be frequently incomplete in the study areas. For this reason, specific medications and dosing, laboratory tests and results are absent from the analysis. Furthermore, self-report is subject to potential recall bias. We therefore select outcomes and interpret results conservatively. We consider the persistence of symptoms suggestive of failure to achieve cure and chronic disease at least in some patients but note that although the elimination of symptoms is of high value to patients, absence of symptoms does not prove cure, since it is also consistent with suppression to latent infection. Furthermore, while we adjust for symptom presence at baseline, symptom persistence could imply the presence of other illnesses or the lasting effects of TB even post-cure. Likewise, we focus on treatment default which is linked to lower TB cure rates in the context of the well-documented selection for resistance that occurs with exposure to TB medication for insufficient duration but purposively avoid definitive conclusions about multidrug resistance status, which we cannot ascertain without laboratory measures.

DOTS regimens employ particular combinations medications for durations that are differentiated for reasons including past TB treatment [73,74]. While our data did not permit a clear delineation of which respondent was on 6 months of Category I DOTS treatment versus 8 months of Category II DOTS treatment, in an analysis confined to individuals previously treated for TB who should be in Category II treatment (see Additional files 12 and 7), we found that treatment discontinuation rates appeared higher among patients who had not completed their prior episodes of TB treatment relative to those who had 45-90 days after the start of treatment and that predictors of treatment discontinuation prior to 8 months were similar to those for prior to 6 months, though power to achieve statistical significance was limited. These findings may be explained in part by the fact that Category II includes an additional, injectable drug (Streptomycin) and a longer intensive phase which might be expected to result in a higher default rate given additional toxicities/side effects (including pain of injections) and inconvenience.

Given that it is commonly believed that at least 6 months of treatment is required to ensure cure in >95% of cases and that the 6-month benchmark is commonly used to assess TB treatment programs, we assessed discontinuation using a logistic regression with discontinuation coded as 1 regardless of whether an individual had completed 1 or 5 months of treatment prior to discontinuing. Alternative survival analyses like those using Cox proportional hazards models can be used to analyze outcomes and may well provide important additional information and power, though mapping from the hazard rate ratios they produce to questions about the likelihood of completing at least 6 months of treatment is substantially less straightforward.

The links between treatment discontinuation and symptom persistence and the mediation of this effect by prior episodes of TB treatment are a complex area of study. Methods like time series analyses that assess evidence of Granger causality along with marginal structural modeling techniques may be useful in further elucidating the nuances of these relationships in future work.

## Conclusions

Premature TB treatment discontinuation remains a substantial problem in 11 districts in Bihar especially among individuals who repeatedly seek TB care. Over 25% of patients have persistent symptoms 25 weeks after treatment initiation; and symptom persistence is more likely for those defaulting early, previously treated and the poor. These are concerning findings given bacteriologic evidence from other studies on the greater likelihood of continued infectiousness among individuals who terminate treatment early and the role of treatment discontinuation in the selection of multidrug resistance. Approaches that reduce treatment discontinuation and symptom persistence should be considered as part of efforts to bolster TB control in settings like Bihar.

## Abbreviations

BEST-TB: Bihar evaluation of social franchising and telemedicine - tuberculosis; DOTS: Directly observed treatment short course; MDR TB: Multidrug resistant tuberculosis; PHCs: Primary health centers; RNTCP: Revised national tuberculosis control programme; SPECT-ERB: Society for the promotion of ethical clinical trials – ethics review board; TB: Tuberculosis.

## Competing interests

Authors have no competing interests to disclose.

## Authors’ contributions

KSB participated in the design of the study, performed the statistical analysis, and helped to draft the manuscript. SS participated in the statistical analysis and helped draft the manuscript. JGF conceived of the study, participated in its design, coordinated and participated in the statistical analysis and helped to draft the manuscript. All authors read and approved the final manuscript.

## Pre-publication history

The pre-publication history for this paper can be accessed here:

http://www.biomedcentral.com/1471-2458/14/418/prepub

## Supplementary Material

Additional file 1: Table S1 Linear Probability Model of Treatment Discontinuation. Click here for file

Additional file 2: Table S2 Likelihood of Treatment Discontinuation <16 Weeks after Treatment Initiation: Logistic Regression Results. Click here for file

Additional file 3: Table S3 Linear Probability Model of Treatment Discontinuation <16 Weeks after Treatment Initiation. Click here for file

Additional file 4: Table S4 Likelihood of Symptom Persistence (Symptoms at 25 Weeks after Treatment Initiation): Logistic Regression Results. Click here for file

Additional file 5: Table S5 Negative Binomial Model of Number of Symptoms Presenting 25 Weeks After Initiating Treatment. Click here for file

Additional file 6: Table S6 Likelihood of Symptom Persistence 16 Weeks after Treatment Initiation: Logistic Regression Results. Click here for file

Additional file 7 Share of Patients Ending Treatment by Category II Treatment Phase. Click here for file

Additional file 8 Case Detection Proportions Reported by RNTCP. Click here for file

Additional file 9 3 Month Case Conversion Rate for Bihar Reported by RNTCP. Click here for file

Additional file 10 Cure Rate Reported by RNTCP. Click here for file

Additional file 11 Default Rates Reported by RNTCP, Bihar. Click here for file

Additional file 12: Table S7 Treatment Default Prior to 8 Months Among Previously Treated Patients. Click here for file

## References

[B1] World Health Organization Global Tuberculosis Control 2011 2011 Geneva: World Health Organization

[B2] Government of India Ministry of Health and Family Welfare: Central TB Division TB India 2011: Revised National TB Control Programme Annual Status Report 2010 New Delhi, India: Government of India Ministry of Health and Family Welfare

[B3] Crofton J Chaulet P Maher D Guidelines for The Management Of Drug-Resistant Tuberculosis 1997 Geneva: World Health Organization

[B4] Heym B Honore N Truffot-Pernot C Banerjee A Schurra C Jacobs WR Jr van Embden JD Grosset JH Cole ST Implications of multidrug resistance for the future of short-course chemotherapy of tuberculosis: a molecular study Lancet 1994 344 8918 293 298 10.1016/S0140-6736(94)91338-2 7914261

[B5] Rowland K Totally Drug-Resistant TB Emerges in India Nature News 2012 2012

[B6] World Health Organization Drug-Resistant Tuberculosis No At Record Levels 2010 Geneva: World Health Organization

[B7] World Health Organization India: Multidrug-resistant Tuberculosis Profile 2011 Geneva: World Health Organization

[B8] Hanif M Malik S Dhingra VK Acquired drug resistance pattern in tuberculosis cases at the State Tuberculosis Centre, Delhi, India Int J Tuberc Lung D 2009 13 1 74 78 19105882

[B9] Sharma SK Kaushik G Jha B George N Arora SK Prevalence of multidrug-resistant tuberculosis among newly diagnosed cases of sputum-positive pulmonary tuberculosis Indian J Med Res 2011 133 308 311 21441685 PMC3103156

[B10] Andrews JR Gandhi NR Moodley P Shah NS Bohlken L Moll AP Pillay M Friedland G Sturm AW Collaborat TFCR Exogenous reinfection as a cause of multidrug-resistant and extensively drug-resistant tuberculosis in Rural South Africa J Infect Dis 2008 198 11 1582 1589 10.1086/592991 18847372

[B11] Singla R Sarin R Khalid UK Mathuria K Singla N Seven-year DOTS-plus pilot experience in India; results, constraints and issues Int J Tuberc Lung Dis 2009 13 976 981 19723377

[B12] Suen SC Bendavid E Goldhaber-Fiebert JD Disease control implications of India's changing multi-drug resistant tuberculosis epidemic PLoS One 2014 9 3 e89822 10.1371/journal.pone.0089822 24608234 PMC3946521

[B13] Thomas A Ramachandran R Rehaman F Jaggarajamma K Santha T Selvakumar N Krishnan N Mohan NS Sundaram V Wares F Narayanan PR Management of multi-drug resistance tuberculosis in the field: tuberculosis research center experience Indian J Tuberc 2007 54 3 117 124 17886699

[B14] Zhao YL Xu SF Wang LX Chin DP Wang SF Jiang GL Xia H Zhou Y Li Q Ou XC Pang Y Song YY Zhao B Zhang HT He GX Guo J Wang Y National survey of drug-resistant tuberculosis in China New Engl J Med 2012 366 23 2161 2170 10.1056/NEJMoa1108789 22670902

[B15] Controlled Comparison of Two Moxifloxacin Containing Treatment Shortening Regimens in Pulmonary Tuberculosis (REMoxTB) http://clinicaltrials.gov/ct2/show/NCT00864383

[B16] A Controlled Trial of a 4-Month Quinolone-Containing Regimen for the Treatment of Pulmonary http://clinicaltrials.gov/ct2/show/NCT00216385

[B17] Sai Babu B Satyanarayana AVV Venkateshwaralu G Ramakrishna U Vikram P Sahu S Wares F Dewan PK Santosha K Jyoti J Chethana R Neelima T Vinod P Yogesh M Chauhan L Initial default among diagnosed sputum smear-positive pulmonary tuberculosis patients in Andhra Pradesh, India Int J Tuberc Lung D 2008 12 9 1055 1058 18713504

[B18] Jaggarajamma K Sudha G Chandrasekaran V Nirupa C Thomas A Santha T Muniyandi M Narayanan PR Reasons for non-compliance among patients treated under Revised National Tuberculosis Control Programme (RNTCP), Tiruvallur District, South India Indian J Tuberc 2007 54 3 130 135 17886701

[B19] Jha UM Satyanarayana S Dewan PK Chadha S Wares F Sahu S Gupta D Chauhan LS Risk factors for treatment default among re-treatment tuberculosis patients in India PLoS One 2010 5 1 e8873 10.1371/journal.pone.0008873 20111727 PMC2810342

[B20] Mandal PK Mandal A Bhattacharyya SK Comparing the Daily Versus the Intermittent Regimens of the Anti-Tubercular Chemotherapy in the Initial Intensive Phase in Non-HIV, Sputum Positive, Pulmonary Tuberculosis Patients J Clin Diagn Res 2013 7 2 292 295 23542708 10.7860/JCDR/2013/5122.2750 PMC3592295

[B21] Mukherjee A Saha I Sarkar A Chowdhury R Gender differences in notification rates, clinical forms and treatment outcome of tuberculosis patients under the RNTCP Lung India 2012 29 2 120 122 10.4103/0970-2113.95302 22628924 PMC3354483

[B22] Sachdeva KS Satyanarayana S Dewan PK Nair SA Reddy R Kundu D Chadha SS Venkatachalaiah AK Parmar M Chauhan LS Source of previous treatment for re-treatment TB cases registered under the National TB control Programme, India, 2010 PLoS One 2011 6 7 e22061 10.1371/journal.pone.0022061 21814566 PMC3140992

[B23] Srinath S Sharath B Santosha K Chadha SS Roopa S Chander K Wares F Chauhan LS Wilson NC Harries AD Tuberculosis 'retreatment others’: profile and treatment outcomes in the state of Andhra Pradesh, India Int J Tuberc Lung D 2011 15 1 105 109 21276305

[B24] Vijay S Kumar P Chauhan LS Vollepore BH Kizhakkethil UP Rao SG Risk factors associated with default among new smear positive TB patients treated under DOTS in India PLoS One 2010 5 3 3 10.1371/journal.pone.0010043 PMC2850369 20386611

[B25] Udwadia ZF Pinto LM Uplekar MW Tuberculosis management by private practitioners in Mumbai, India: has anything changed in two decades? PLoS One 2010 5 8 e12023 10.1371/journal.pone.0012023 20711502 PMC2918510

[B26] Uplekar MW Shepard DS Treatment of tuberculosis by private general practitioners in India Tubercle 1991 72 4 284 290 10.1016/0041-3879(91)90055-W 1811360

[B27] Vandan N Ali M Prasad R Kuroiwa C Assessment of doctors’ knowledge regarding tuberculosis management in Lucknow, India: a public-private sector comparison Public Health 2009 123 484 489 10.1016/j.puhe.2009.05.004 19560176

[B28] International Institute for Population Sciences Reproductive and Child Health: District Level Household Survey (DLHS-2), 2002-04 2006 Mumbai, India: International Institute for Population Sciences

[B29] International Institute for Population Sciences (IIPS) District Level Household and Facility Survey (DLHS-3), 2007-08 2010 Mumbai, India: International Institute for Population Sciences

[B30] Government of India Ministry of Health and Family Welfare: Central TB Division TB India 2012: Revised National TB Control Programme annual status repor 2012 New Delhi: Ministry of Health and Family Welfare, Central TB Division

[B31] Barat D Kumar G Prevalence and pattern of acquired drug resistance in tuberculosis including MDR-TB in Bihar Assoc Physicians India 2003 51 327 12839372

[B32] Gandhi NR Nunn P Dheda K Schaaf HS Zignol M van Soolingen D Jensen P Bayona J Multidrug-resistant and extensively drug-resistant tuberculosis: a threat to global control of tuberculosis Lancet 2010 375 9728 1830 1843 10.1016/S0140-6736(10)60410-2 20488523

[B33] Wright A Zignol M Van Deun A Falzon D Gerdes SR Feldman K Hoffner S Drobniewski F Barrera L van Soolingen D Boulabhal F Paramasivan CN Kam KM Mitarai S Nunn P Raviglione M Epidemiology of antituberculosis drug resistance 2002-07: an updated analysis of the Global Project on Anti-Tuberculosis Drug Resistance Surveillance Lancet 2009 373 9678 1861 1873 10.1016/S0140-6736(09)60331-7 19375159

[B34] Collaborations for Health System Improvement and Impact Evaluation in India http://www.cohesiveindia.org/research-projects.html#best

[B35] Chadha VK Kumar P Anjinappa SM Singh S Narasimhaiah S Joshi MV Gupta J Lakshminarayana Ramchandra J Velu M Papkainathan S Babu S Krishna H Prevalence of pulmonary tuberculosis among adults in a rural sub-district of South India PLoS One 2012 7 8 e42625 10.1371/journal.pone.0042625 22956993 PMC3431961

[B36] Kolappan C Subramani R Chandrasekaran V Thomas A Trend in tuberculosis infection prevalence in a rural area in South India after implementation of the DOTS strategy Int J Tuberc Lung Dis 2012 16 10 1315 1319 10.5588/ijtld.12.0098 23107632

[B37] Oxlade O Murray M Tuberculosis and poverty: why are the poor at greater risk in India? PLoS One 2012 7 11 e47533 10.1371/journal.pone.0047533 23185241 PMC3501509

[B38] Rao VG Bhat J Yadav R Gopalan GP Nagamiah S Bhondeley MK Anjinappa SM Ramchandra J Chadha VK Wares F Prevalence of pulmonary tuberculosis–a baseline survey in central India PLoS One 2012 7 8 e43225 10.1371/journal.pone.0043225 22952651 PMC3430677

[B39] Satyanarayana S Nair SA Chadha SS Shivashankar R Sharma G Yadav S Mohanty S Kamineni V Wilson NC Harries AD Dewan PK From where are tuberculosis patients accessing treatment in India? results from a cross-sectional community based survey of 30 districts PLoS One 2011 6 9 e24160 10.1371/journal.pone.0024160 21912669 PMC3166304

[B40] Corbett EL Zezai A Cheung YB Bandason T Dauya E Munyati SS Butterworth AE Rusikaniko S Churchyard GJ Mungofa S Hayes RJ Mason PR Provider-initiated symptom screening for tuberculosis in Zimbabwe: diagnostic value and the effect of HIV status Bull World Health Organ 2010 88 1 13 21 10.2471/BLT.08.055467 20428349 PMC2802433

[B41] van der Werf MJ Enarson DA Borgdorff MW How to identify tuberculosis cases in a prevalence survey Int J Tuberc Lung D 2008 12 11 1255 1260 18926034

[B42] The World Health Organization Multidrug and extensively drug-resistant TB (M/XDR-TB): 2010 global report on surveillance and response 2010 The World Health Organization: Geneva

[B43] Charles N Thomas B Watson B Raja Sakthivel M Chandrasekeran V Wares F Care seeking behavior of chest symptomatics: a community based study done in South India after the implementation of the RNTCP PLoS One 2010 5 9 e12379 10.1371/journal.pone.0012379 20862219 PMC2942833

[B44] Dhingra VK Rajpal S Taneja DK Kalra D Malhotra R Health care seeking pattern of tuberculosis patients attending an urban TB clinic in Delhi J Commun Dis 2002 34 3 185 192 14703053

[B45] Dye C The potential impact of new diagnostic tests on tuberculosis epidemics Indian J Med Res 2012 135 5 737 744 22771607 PMC3401708

[B46] Kapoor SK Raman AV Sachdeva KS Satyanarayana S How did the TB patients reach DOTS services in Delhi? A study of patient treatment seeking behavior PLoS One 2012 7 8 e42458 10.1371/journal.pone.0042458 22879990 PMC3412865

[B47] Pantoja A Lonnroth K Lal SS Chauhan LS Uplekar M Padma MR Unnikrishnan KP Rajesh J Kumar P Sahu S Wares F Floyd K Economic evaluation of public-private mix for tuberculosis care and control, India. Part II. Cost and cost-effectiveness Int J Tuberc Lung Dis 2009 13 6 705 712 19460245

[B48] Pradhan A Kielmann K Gupte H Bamne A Porter JD Rangan S What 'outliers’ tell us about missed opportunities for tuberculosis control: a cross-sectional study of patients in Mumbai, India BMC Public Health 2010 10 263 10.1186/1471-2458-10-263 20482899 PMC2887819

[B49] Rajeswari R Chandrasekaran V Suhadev M Sivasubramaniam S Sudha G Renu G Factors associated with patient and health system delays in the diagnosis of tuberculosis in South India Int J Tuberc Lung Dis 2002 6 9 789 795 12234134

[B50] Sudha G Nirupa C Rajasakthivel M Sivasusbramanian S Sundaram V Bhatt S Subramaniam K Thiruvalluvan E Mathew R Renu G Santha T Factors influencing the care-seeking behaviour of chest symptomatics: a community-based study involving rural and urban population in Tamil Nadu, South India Trop Med Int Health 2003 8 4 336 341 10.1046/j.1365-3156.2003.01010.x 12667153

[B51] Tamhane A Ambe G Vermund SH Kohler CL Karande A Sathiakumar N Pulmonary tuberculosis in Mumbai, India: factors responsible for patient and treatment delays Int J Prev Med 2012 3 8 569 580 22973488 PMC3429805

[B52] Mauch V Bonsu F Gyapong M Awini E Suarez P Marcelino B Melgen RE Lonnroth K Nhung NV Hoa NB Klinkenberg E Free tuberculosis diagnosis and treatment are not enough: patient cost evidence from three continents Int J Tuberc Lung Dis 2013 17 3 381 387 10.5588/ijtld.12.0368 23407227

[B53] Dye C Lonnroth K Jaramillo E Williams BG Raviglione M Trends in tuberculosis incidence and their determinants in 134 countries Bull World Health Organ 2009 87 9 683 691 10.2471/BLT.08.058453 19784448 PMC2739916

[B54] Goldhaber-Fiebert JD Jeon CY Cohen T Murray MB Diabetes mellitus and tuberculosis in countries with high tuberculosis burdens: individual risks and social determinants Int J Epidemiol 2011 40 2 417 428 10.1093/ije/dyq238 21252210 PMC3621385

[B55] Rasanathan K Sivasankara Kurup A Jaramillo E Lonnroth K The social determinants of health: key to global tuberculosis control Int J Tuberc Lung Dis 2011 15 Suppl 2 S30 36 21740657 10.5588/ijtld.10.0691

[B56] Filmer D Pritchett LH Estimating wealth effects without expenditure data - Or tears: an application to educational enrollments in states of India Demography 2001 38 1 115 132 11227840 10.1353/dem.2001.0003

[B57] International Institute for Population Sciences (IIPS) and Macro International National Family Health Survey (NFHS-3) 2005-06, India: Key Findings 2007 Mumbai: IIPS

[B58] Muniyandi M Ramachandran R Gopi PG Chandrasekaran V Subramani R Sadacharam K Kumaran P Santha T Wares F Narayanan PR The prevalence of tuberculosis in different economic strata: a community survey from South India Int J Tuberc Lung Dis 2007 11 9 1042 1045 17705985

[B59] StataCorp Stata Survey Data Reference Manual, Release 13 2013 College Station, TX: StataCorp LP

[B60] Malmborg R Mann G Thomson R Squire SB Can public-private collaboration promote tuberculosis case detection among the poor and vulnerable? Bull World Health Organ 2006 84 9 752 758 17128346 PMC2627476

[B61] Pantoja A Floyd K Unnikrishnan KP Jitendra R Padma MR Lal SS Uplekar M Chauhan LS Kumar P Sahu S Wares F Lönnroth K Economic evaluation of public-private mix for tuberculosis care and control, India. Part I. Socio-economic profile and costs among tuberculosis patients Int J Tuberc Lung Dis 2009 13 6 698 704 19460244

[B62] Udwadia ZF Pinto LM Uplekar MW Tuberculosis management by private practitioners in Mumbai, India: has anything changed in two decades? PLoS One 2010 5 8 e12023 10.1371/journal.pone.0012023 20711502 PMC2918510

[B63] Lonnroth K Aung T Maung W Kluge H Uplekar M Social franchising of TB care through private GPs in Myanmar: an assessment of treatment results, access, equity and financial protection Health Policy Plann 2007 22 3 156 166 10.1093/heapol/czm007 17434870

[B64] Munro SA Lewin SA Smith HJ Engel ME Fretheim A Volmink J Patient adherence to tuberculosis treatment: a systematic review of qualitative research PLoS Med 2007 4 7 e238 10.1371/journal.pmed.0040238 17676945 PMC1925126

[B65] Kliiman K Altraja A Predictors and mortality associated with treatment default in pulmonary tuberculosis Int J Tuberc Lung Dis 2010 14 4 454 463 20202304

[B66] Garrido Mda S Penna ML Perez-Porcuna TM de Souza AB Marreiro Lda S Albuquerque BC Martinez-Espinosa FE Buhrer-Sekula S Factors associated with tuberculosis treatment default in an endemic area of the Brazilian Amazon: a case control-study PLoS One 2012 7 6 e39134 10.1371/journal.pone.0039134 22720052 PMC3373579

[B67] Central TB Division DGoHS, Ministry of Health and Family Welfare TB India 2006, RNTCP Status Report 2006 New Delhi

[B68] Central TB Division DGoHS, Ministry of Health and Family Welfare TB India 2007, RNTCP Status Report 2007 New Delhi

[B69] Central TB Division DGoHS, Ministry of Health and Family Welfare TB India 2008, RNTCP Status Report 2008 New Delhi

[B70] Central TB Division DGoHS, Ministry of Health and Family Welfare TB India 2009, RNTCP Status Report 2009 New Delhi

[B71] Central TB Division DGoHS, Ministry of Health and Family Welfare TB India 2010, RNTCP Status Report 2010 New Delhi

[B72] Central TB Division DGoHS, Ministry of Health and Family Welfare TB India 2011, Revised National TB COntrol Programme Annual Status Report 2011 New Delhi

[B73] Chauhan LS Agarwal SP Agarwal SP, Chauhan LS The Revised National Tuberculosis Control Programme 23 Tuberculosis Control in India 2005 Delhi: Central TB Division, Ministry of Health and Family Welfare, Directorate General of Health Services

[B74] World Health Organization Tuberculosis guidelines, Fourth edition 2010 Geneva: The World Health Organization

