# Supplementary material for: Tuberculosis treatment discontinuation and symptom persistence: an observational study of Bihar, India’s public care system covering >100,000,000 inhabitants
Source: BMC Public Health. 2014 May 1;14:418. doi: 10.1186/1471-2458-14-418 (PMC4041057; doi:10.1186/1471-2458-14-418)
Supplement: Additional file 1: Table S1 — Linear Probability Model of Treatment Discontinuation. [file 1471-2458-14-418-S1.docx]

**Additional file 1: Table S1: Linear Probability Model of Treatment Discontinuation**

|  | | **Univariate Regression** | | **Multivariate Regression** | | | | | |
| --- | --- | --- | --- | --- | --- | --- | --- | --- | --- |
|  | | **All Patients** | | **All Patients** | | **Patients with prior TB** | | **Patients with no prior TB** | |
|  | | **OR** | **(95% CI)** | **OR** | **(95% CI)** | **OR** | **(95% CI)** | **OR** | **(95% CI)** |
| **Prior TB Status** | | | | | | | | | |
| **Prior TB Treatment Episode** | 0.40* | | (0.23 - 0.58) | 0.33* | (0.15 - 0.50) |  |  |  |  |
| **Prior TB & Completed Prior Treatment** | -0.34* | | (-0.53 - -0.15) | -0.31* | (-0.50 - -0.13) | -0.18 | (-0.37 - 0.00) |  |  |
|  |  | |  |  |  |  |  |  |  |
| **Current Illness Treatment and Illness Characteristics** | | | | | | | | | |
| **Total Weeks from Symptom Onset to Treatment Initiation** | 0.00 | | (-0.01 - 0.01) | 0.00 | (-0.01 - 0.01) | 0.00 | (-0.01 - 0.02) | 0.00 | (-0.00 - 0.01) |
| **Number of Providers Visited** | 0.31* | | (0.20 - 0.42) | 0.25* | (0.13 - 0.37) | 0.18 | (-0.01 - 0.37) | 0.34* | (0.17 - 0.50) |
| **Treatment or Medication Fees** | 0.29* | | (0.12 - 0.46) | 0.21* | (0.05 - 0.37) | -0.05 | (-0.53 - 0.42) | 0.21* | (0.00 - 0.42) |
| **Travel Costs** | 0.13* | | (0.02 - 0.23) | 0.11* | (0.01 - 0.21) | -0.02 | (-0.42 - 0.37) | 0.15* | (0.05 - 0.24) |
| **Treatment, Medication and Travel Costs** | -0.23* | | (-0.42 - -0.04) | -0.14 | (-0.32 - 0.04) | 0.05 | (-0.40 - 0.51) | -0.18 | (-0.42 - 0.06) |
| **2 or Fewer Symptoms at Treatment Initiation**** | 0.03 | | (-0.05 - 0.10) | 0.08* | (0.00 - 0.15) | 0.11 | (-0.04 - 0.27) | 0.04 | (-0.04 - 0.12) |
| **3-4 Symptoms at Treatment Initiation**** | 0.05 | | (-0.05 - 0.16) | 0.07 | (-0.03 - 0.17) | 0.12 | (-0.05 - 0.28) | 0.02 | (-0.07 - 0.11) |
|  |  | |  |  |  |  |  |  |  |
| **Patient and Household Characteristics** | | | | | | | | | |
| **Male** | 0.04 | | (-0.03 - 0.11) | 0.04 | (-0.03 - 0.12) | 0.01 | (-0.11 - 0.12) | 0.04 | (-0.03 - 0.11) |
| **Age** | -0.01 | | (-0.02 - 0.00) | -0.01 | (-0.02 - 0.00) | -0.02 | (-0.04 - 0.00) | -0.00 | (-0.01 - 0.00) |
| **Age Squared** | 0.00 | | (-0.00 - 0.00) | 0.00 | (-0.00 - 0.00) | 0.00 | (-0.00 - 0.00) | 0.00 | (-0.00 - 0.00) |
| **Education** | 0.00 | | (-0.01 - 0.01) | -0.00 | (-0.01 - 0.01) | -0.02* | (-0.04 - -0.00) | 0.00 | (-0.01 - 0.01) |
| **Hindu** | -0.03 | | (-0.12 - 0.06) | -0.03 | (-0.12 - 0.06) | -0.15 | (-0.33 - 0.03) | -0.00 | (-0.10 - 0.09) |
| **Scheduled Caste, Tribe, Other Backwards Class** | -0.02 | | (-0.12 - 0.07) | -0.02 | (-0.12 - 0.07) | -0.19 | (-0.41 - 0.03) | 0.01 | (-0.09 - 0.11) |
| **Number of Kids** | -0.01 | | (-0.03 - 0.01) | 0.00 | (-0.02 - 0.03) | -0.02 | (-0.06 - 0.03) | 0.01 | (-0.02 - 0.04) |
| **Household Size** | -0.01 | | (-0.02 - 0.01) | -0.01 | (-0.03 - 0.01) | 0.02 | (-0.02 - 0.05) | -0.01 | (-0.03 - 0.01) |
| **Poor** | 0.01 | | (-0.07 - 0.10) | -0.01 | (-0.10 - 0.09) | -0.03 | (-0.20 - 0.14) | 0.04 | (-0.05 - 0.12) |
| **Middle Income** | -0.06 | | (-0.17 - 0.05) | -0.02 | (-0.12 - 0.07) | 0.04 | (-0.10 - 0.18) | -0.01 | (-0.08 - 0.07) |
|  |  | |  |  |  |  |  |  |  |
| **Observations** | 1007 | | | 1007 | | 196 | | 811 | |

* p<0.05

** Comparator group is ≥5 Symptoms at Treatment Initiation
