# Supplementary material for: Tuberculosis treatment discontinuation and symptom persistence: an observational study of Bihar, India’s public care system covering >100,000,000 inhabitants
Source: BMC Public Health. 2014 May 1;14:418. doi: 10.1186/1471-2458-14-418 (PMC4041057; doi:10.1186/1471-2458-14-418)
Supplement: Additional file 2: Table S2 — Likelihood of Treatment Discontinuation <16 Weeks after Treatment Initiation: Logistic Regression Results. [file 1471-2458-14-418-S2.docx]

**Additional file 2: Table S2: Likelihood of Treatment Discontinuation <16 Weeks after Treatment Initiation: Logistic Regression Results**

|  | **Univariate Regression** | | **Multivariate Regression** | | | | | |
| --- | --- | --- | --- | --- | --- | --- | --- | --- |
|  | **All Patients** | | **All Patients** | | **Patients with prior TB** | | **Patients with no prior TB** | |
|  | **OR** | **(95% CI)** | **OR** | **(95% CI)** | **OR** | **(95% CI)** | **OR** | **(95% CI)** |
|  |  |  |  |  |  |  |  |  |
| **Prior TB Status** |  |  |  |  |  |  |  |  |
| **Prior TB Treatment Episode** | 6.75* | (2.85 - 16.02) | 4.63* | (1.88 - 11.42) |  |  |  |  |
| **Prior TB & completed treatment prior treatment** | 0.31* | (0.12 - 0.82) | 0.26* | (0.09 - 0.72) | 0.17* | (0.04 - 0.78) |  |  |
|  |  |  |  |  |  |  |  |  |
| **Current Illness Treatment and Illness Characteristics** |  |  |  |  |  |  |  |  |
| **Total Delay from Symptom Onset to Treatment Initiation** | 0.98 | (0.92 - 1.05) | 0.99 | (0.93 - 1.06) | 1.07 | (0.90 - 1.27) | 1.01 | (0.93 - 1.09) |
| **Number of Providers Visited** | 6.39* | (2.78 - 14.67) | 5.60* | (2.18 - 14.37) | 2.43 | (0.18 - 33.45) | 14.00* | (5.02 - 39.01) |
| **Treatment or Medication Fees** | 4.63 | (0.98 - 21.93) | 1.88 | (0.36 - 9.92) | 1.49 | (0.09 - 25.66) | 1.64 | (0.26 - 10.37) |
| **Travel Costs** | 1.72 | (0.53 - 5.52) | 1.50 | (0.49 - 4.59) | 0.20 | (0.01 - 3.35) | 3.34 | (0.89 - 12.55) |
| **Treatment, Medication and Travel Costs** | 0.50 | (0.11 - 2.26) | 1.13 | (0.22 - 5.78) | 7.04 | (0.16 - 303.25) | 0.73 | (0.11 - 5.04) |
|  |  |  |  |  |  |  |  |  |
| **2 or Fewer Symptoms at Treatment Initiation**** | 0.78 | (0.42 - 1.42) | 1.08 | (0.56 - 2.09) | 1.41 | (0.34 - 5.87) | 0.92 | (0.44 - 1.93) |
| **3-4 Symptoms at Treatment Initiation**** | 0.98 | (0.45 - 2.13) | 1.04 | (0.49 - 2.21) | 2.24 | (0.45 - 11.17) | 0.65 | (0.29 - 1.45) |
|  |  |  |  |  |  |  |  |  |
| **Patient and Household Characteristics** |  |  |  |  |  |  |  |  |
| **Male** | 1.09 | (0.68 - 1.77) | 1.13 | (0.66 - 1.93) | 1.29 | (0.47 - 3.56) | 1.00 | (0.56 - 1.78) |
| **Age** | 0.94* | (0.89 - 0.99) | 0.94* | (0.89 - 1.00) | 0.89 | (0.72 - 1.10) | 0.95 | (0.89 - 1.01) |
| **Age Squared** | 1.00* | (1.00 - 1.00) | 1.00 | (1.00 - 1.00) | 1.00 | (1.00 - 1.00) | 1.00 | (1.00 - 1.00) |
| **Education** | 0.99 | (0.93 - 1.06) | 0.97 | (0.91 - 1.04) | 0.78* | (0.62 - 0.98) | 1.00 | (0.93 - 1.07) |
| **Hindu** | 0.67 | (0.35 - 1.26) | 0.71 | (0.36 - 1.42) | 0.11* | (0.02 - 0.55) | 1.03 | (0.44 - 2.44) |
| **Scheduled Caste, Tribe, Other Backwards Class** | 0.63 | (0.35 - 1.14) | 0.58 | (0.28 - 1.20) | 0.60 | (0.06 - 6.45) | 0.58 | (0.25 - 1.35) |
| **Number of Kids** | 0.97 | (0.79 - 1.18) | 0.97 | (0.78 - 1.20) | 0.71 | (0.45 - 1.11) | 1.04 | (0.80 - 1.35) |
| **Household Size** | 1.02 | (0.90 - 1.16) | 1.03 | (0.90 - 1.19) | 1.46 | (1.00 - 2.13) | 0.96 | (0.82 - 1.13) |
| **Poor** | 1.02 | (0.55 - 1.87) | 0.93 | (0.48 - 1.81) | 0.73 | (0.12 - 4.55) | 1.32 | (0.62 - 2.82) |
| **Middle Income** | 0.54 | (0.23 - 1.27) | 0.70 | (0.36 - 1.34) | 0.44 | (0.11 - 1.73) | 0.89 | (0.50 - 1.59) |
|  |  |  |  |  |  |  |  |  |
| **Observations** | 1007 | | 1007 | | 196 | | 811 | |

* p<0.05

** Comparator group is ≥5 Symptoms at Treatment Initiation
