# Supplementary material for: Tuberculosis treatment discontinuation and symptom persistence: an observational study of Bihar, India’s public care system covering >100,000,000 inhabitants
Source: BMC Public Health. 2014 May 1;14:418. doi: 10.1186/1471-2458-14-418 (PMC4041057; doi:10.1186/1471-2458-14-418)
Supplement: Additional file 3: Table S3 — Linear Probability Model of Treatment Discontinuation <16 Weeks after Treatment Initiation. [file 1471-2458-14-418-S3.docx]

**Additional file 3: Table S3: Linear Probability Model of Treatment Discontinuation <16 Weeks after Treatment Initiation**

|  | **Univariate Regression** | | **Multivariate Regression** | | | | | |
| --- | --- | --- | --- | --- | --- | --- | --- | --- |
|  | **All Patients** | | **All Patients** | | **Patients with prior TB** | | **Patients with no prior TB** | |
|  |  | **(95% CI)** |  | **(95% CI)** |  | **(95% CI)** |  | **(95% CI)** |
| **Prior TB Status** |  |  |  |  |  |  |  |  |
| **Prior TB Treatment Episode** | 0.36* | (0.18 - 0.55) | 0.29* | (0.11 - 0.47) |  |  |  |  |
| **Prior TB & completed treatment prior treatment** | -0.27* | (-0.47 - -0.07) | -0.26* | (-0.45 - -0.07) | -0.17* | (-0.34 - -0.01) |  |  |
|  |  |  |  |  |  |  |  |  |
| **Current Illness Treatment and Illness Characteristics** |  |  |  |  |  |  |  |  |
| **Total Delay from Symptom Onset to Treatment Initiation** | -0.00 | (-0.01 - 0.01) | -0.00 | (-0.01 - 0.01) | 0.00 | (-0.01 - 0.02) | 0.00 | (-0.01 - 0.01) |
| **Number of Providers Visited** | 0.30* | (0.17 - 0.44) | 0.27* | (0.12 - 0.41) | 0.12 | (-0.07 - 0.31) | 0.43* | (0.25 - 0.60) |
| **Treatment or Medication Fees** | 0.16 | (-0.00 - 0.32) | 0.05 | (-0.10 - 0.20) | -0.03 | (-0.51 - 0.45) | 0.01 | (-0.11 - 0.13) |
| **Travel Costs** | 0.05 | (-0.05 - 0.14) | 0.03 | (-0.05 - 0.12) | -0.18 | (-0.58 - 0.22) | 0.08* | (0.00 - 0.16) |
| **Treatment, Medication and Travel Costs** | -0.06 | (-0.22 - 0.10) | 0.04 | (-0.12 - 0.19) | 0.20 | (-0.29 - 0.68) | 0.00 | (-0.13 - 0.14) |
|  |  |  |  |  |  |  |  |  |
| **2 or Fewer Symptoms at Treatment Initiation**** | -0.03 | (-0.10 - 0.04) | 0.01 | (-0.06 - 0.08) | 0.00 | (-0.13 - 0.14) | -0.01 | (-0.08 - 0.06) |
| **3-4 Symptoms at Treatment Initiation**** | 0.00 | (-0.10 - 0.10) | 0.01 | (-0.08 - 0.11) | 0.07 | (-0.08 - 0.23) | -0.04 | (-0.11 - 0.03) |
|  |  |  |  |  |  |  |  |  |
| **Patient and Household Characteristics** |  |  |  |  |  |  |  |  |
| **Male** | 0.01 | (-0.05 - 0.07) | 0.02 | (-0.04 - 0.08) | 0.02 | (-0.08 - 0.12) | 0.00 | (-0.05 - 0.05) |
| **Age** | -0.01 | (-0.02 - 0.00) | -0.01* | (-0.01 - -0.00) | -0.01 | (-0.03 - 0.01) | -0.00 | (-0.01 - 0.00) |
| **Age Squared** | 0.00 | (-0.00 - 0.00) | 0.00* | (0.00 - 0.00) | 0.00 | (-0.00 - 0.00) | 0.00 | (-0.00 - 0.00) |
| **Education** | -0.00 | (-0.01 - 0.01) | -0.00 | (-0.01 - 0.00) | -0.02* | (-0.03 - -0.00) | -0.00 | (-0.01 - 0.01) |
| **Hindu** | -0.05 | (-0.14 - 0.04) | -0.03 | (-0.12 - 0.05) | -0.20* | (-0.39 - -0.01) | 0.00 | (-0.08 - 0.08) |
| **Scheduled Caste, Tribe, Other Backwards Class** | -0.06 | (-0.14 - 0.02) | -0.06 | (-0.15 - 0.03) | -0.13 | (-0.40 - 0.13) | -0.05 | (-0.14 - 0.04) |
| **Number of Kids** | -0.00 | (-0.03 - 0.02) | -0.00 | (-0.03 - 0.02) | -0.03 | (-0.07 - 0.02) | 0.00 | (-0.02 - 0.02) |
| **Household Size** | 0.00 | (-0.01 - 0.02) | 0.00 | (-0.01 - 0.02) | 0.03 | (-0.01 - 0.06) | -0.00 | (-0.02 - 0.01) |
| **Poor** | 0.00 | (-0.08 - 0.08) | -0.01 | (-0.09 - 0.07) | 0.00 | (-0.16 - 0.17) | 0.03 | (-0.04 - 0.10) |
| **Middle Income** | -0.07 | (-0.17 - 0.03) | -0.04 | (-0.12 - 0.04) | -0.02 | (-0.16 - 0.12) | -0.01 | (-0.06 - 0.04) |
|  |  |  |  |  |  |  |  |  |
| **Observations** | 1007 | | 1007 | | 196 | | 811 | |

* p<0.05

** Comparator group is ≥5 Symptoms at Treatment Initiation
