# Supplementary material for: Tuberculosis treatment discontinuation and symptom persistence: an observational study of Bihar, India’s public care system covering >100,000,000 inhabitants
Source: BMC Public Health. 2014 May 1;14:418. doi: 10.1186/1471-2458-14-418 (PMC4041057; doi:10.1186/1471-2458-14-418)
Supplement: Additional file 4: Table S4 — Likelihood of Symptom Persistence (Symptoms at 25 Weeks after Treatment Initiation): Logistic Regression Results. [file 1471-2458-14-418-S4.docx]

**Additional file 4: Table S4: Likelihood of Symptom Persistence (Symptoms at 25 Weeks after Treatment Initiation): Logistic Regression Results**

|  | **Univariate Regression** | | **Multivariate Regression** | | | | | |
| --- | --- | --- | --- | --- | --- | --- | --- | --- |
|  |  |  | **All Patients** | | **Patients with prior TB** | | **Patients with no prior TB** | |
|  | **OR** | **(95% CI)** | **OR** | **(95% CI)** | **OR** | **(95% CI)** | **OR** | **(95% CI)** |
| **Prior TB Status** |  |  |  |  |  |  |  |  |
| **Prior TB Treatment Episode** | 4.59* | (2.17 - 9.70) | 5.05* | (1.90 - 13.38) |  |  |  |  |
| **Prior TB & completed treatment prior treatment** | 1.63 | (0.58 - 4.62) | 2.40 | (0.78 - 7.37) | 0.81 | (0.26 - 2.48) |  |  |
|  |  |  |  |  |  |  |  |  |
| **Current Illness Treatment and Illness Characteristics** |  |  |  |  |  |  |  |  |
| **Total Delay from Symptom Onset to Treatment Initiation** | 1.01 | (0.97 - 1.05) | 0.96 | (0.91 - 1.01) | 0.95 | (0.86 - 1.05) | 0.96 | (0.89 - 1.02) |
| **Number of Providers Visited** | 2.73* | (1.42 - 5.25) | 2.06 | (0.93 - 4.56) | 3.76* | (1.00 - 14.11) | 1.10 | (0.25 - 4.86) |
| **Treatment or Medication Fees** | 2.79* | (1.16 - 6.71) | 1.17 | (0.42 - 3.25) | 1.30 | (0.12 - 14.46) | 1.59 | (0.43 - 5.92) |
| **Travel Costs** | 2.16* | (1.10 - 4.27) | 1.89 | (0.91 - 3.92) | 0.73 | (0.06 - 8.66) | 1.74 | (0.81 - 3.70) |
| **Treatment, Medication and Travel Costs** | 0.13* | (0.05 - 0.33) | 0.16* | (0.06 - 0.46) | 1.30 | (0.08 - 22.00) | 0.10* | (0.03 - 0.39) |
| **2 or Fewer Symptoms at Treatment Initiation**** | 0.95 | (0.57 - 1.58) | 1.10 | (0.64 - 1.89) | 0.89 | (0.28 - 2.84) | 1.93 | (0.96 - 3.88) |
| **3-4 Symptoms at Treatment Initiation**** | 0.57 | (0.28 - 1.16) | 0.50 | (0.18 - 1.43) | 0.42 | (0.12 - 1.45) | 1.11 | (0.37 - 3.35) |
|  |  |  |  |  |  |  |  |  |
| **Treated < 8 Weeks** | 1.59 | (0.73 - 3.43) | 0.50 | (0.19 - 1.35) | 0.35 | (0.08 - 1.55) | 0.70 | (0.17 - 2.89) |
| **Treated 9-16 Weeks** | 0.90 | (0.31 - 2.61) | 0.90 | (0.29 - 2.76) | 0.29 | (0.07 - 1.17) | 2.12 | (0.75 - 6.02) |
|  |  |  |  |  |  |  |  |  |
| **Patient and Household Characteristics** |  |  |  |  |  |  |  |  |
| **Male** | 0.66* | (0.45 - 0.96) | 0.56* | (0.33 - 0.93) | 0.41 | (0.15 - 1.12) | 0.76 | (0.45 - 1.30) |
| **Age** | 0.97 | (0.92 - 1.01) | 0.97 | (0.91 - 1.03) | 0.97 | (0.88 - 1.07) | 0.96 | (0.90 - 1.03) |
| **Age Squared** | 1.00* | (1.00 - 1.00) | 1.00 | (1.00 - 1.00) | 1.00 | (1.00 - 1.00) | 1.00 | (1.00 - 1.00) |
| **Education** | 0.95 | (0.91 - 1.00) | 1.02 | (0.95 - 1.09) | 0.99 | (0.87 - 1.13) | 0.99 | (0.92 - 1.06) |
| **Hindu** | 0.65 | (0.41 - 1.03) | 0.54 | (0.27 - 1.08) | 0.61 | (0.10 - 3.94) | 0.49 | (0.22 - 1.08) |
| **Scheduled Caste, Tribe, Other Backwards Class** | 0.85 | (0.52 - 1.38) | 0.90 | (0.51 - 1.59) | 0.71 | (0.09 - 5.56) | 0.92 | (0.46 - 1.86) |
| **Number of Kids** | 1.02 | (0.88 - 1.19) | 1.03 | (0.84 - 1.28) | 0.78 | (0.54 - 1.12) | 1.14 | (0.88 - 1.49) |
| **Household Size** | 1.06 | (0.96 - 1.17) | 0.99 | (0.87 - 1.13) | 1.18 | (0.89 - 1.57) | 0.90 | (0.77 - 1.06) |
| **Poor** | 2.37* | (1.44 - 3.91) | 2.94* | (1.51 - 5.72) | 1.89 | (0.66 - 5.38) | 2.91* | (1.49 - 5.68) |
| **Middle Income** | 0.86 | (0.45 - 1.63) | 1.02 | (0.52 - 2.00) | 0.81 | (0.24 - 2.73) | 0.84 | (0.48 - 1.47) |
|  |  |  |  |  |  |  |  |  |
| **Observations** | 1007 | | 1007 | | 196 | | 811 | |

* p<0.05

** Comparator group is ≥5 Symptoms at Treatment Initiation

|  | **Univariate Regression** | | **Multivariate Regression** | | | | | |
| --- | --- | --- | --- | --- | --- | --- | --- | --- |
|  |  |  | **All Patients** | | **Patients with prior TB** | | **Patients with no prior TB** | |
|  | **OR** | **(95% CI)** | **OR** | **(95% CI)** | **OR** | **(95% CI)** | **OR** | **(95% CI)** |
| **Prior TB Status** |  |  |  |  |  |  |  |  |
| **Prior TB Treatment Episode** | 4.59* | (2.17 - 9.70) | 5.01* | (1.84 - 13.64) |  |  |  |  |
| **Prior TB & completed treatment prior treatment** | 1.63 | (0.58 - 4.62) | 2.37 | (0.76 - 7.41) | 0.83 | (0.26 - 2.64) |  |  |
|  |  |  |  |  |  |  |  |  |
| **Current Illness Treatment and Illness Characteristics** |  |  |  |  |  |  |  |  |
| **Total Delay from Symptom Onset to Treatment Initiation** | 1.01 | (0.97 - 1.05) | 0.96 | (0.91 - 1.01) | 0.95 | (0.86 - 1.05) | 0.95 | (0.89 - 1.02) |
| **Number of Providers Visited** | 2.73* | (1.42 - 5.25) | 1.81 | (0.81 - 4.06) | 4.09* | (1.05 - 15.93) | 0.84 | (0.21 - 3.38) |
| **Treatment or Medication Fees** | 2.79* | (1.16 - 6.71) | 1.22 | (0.44 - 3.38) | 1.14 | (0.09 - 13.81) | 1.54 | (0.41 - 5.86) |
| **Travel Costs** | 2.16* | (1.10 - 4.27) | 1.89 | (0.92 - 3.88) | 0.79 | (0.06 - 10.29) | 1.67 | (0.78 - 3.61) |
| **Treatment, Medication and Travel Costs** | 0.13* | (0.05 - 0.33) | 0.16* | (0.06 - 0.45) | 1.27 | (0.07 - 23.11) | 0.10* | (0.03 - 0.41) |
| **2 or Fewer Symptoms at Treatment Initiation**** | 0.95 | (0.57 - 1.58) | 1.11 | (0.64 - 1.93) | 0.96 | (0.32 - 2.91) | 1.77 | (0.86 - 3.64) |
| **3-4 Symptoms at Treatment Initiation**** | 0.57 | (0.28 - 1.16) | 0.51 | (0.18 - 1.44) | 0.44 | (0.14 - 1.40) | 1.03 | (0.33 - 3.21) |
|  |  |  |  |  |  |  |  |  |
| **Completed At Least 25 Weeks of Treatment** | 0.99 | (0.53 - 1.82) | 1.25 | (0.63 - 2.48) | 2.72* | (1.04 - 7.14) | 0.69 | (0.37 - 1.30) |
|  |  |  |  |  |  |  |  |  |
| **Patient and Household Characteristics** |  |  |  |  |  |  |  |  |
| **Male** | 0.66* | (0.45 - 0.96) | 0.57* | (0.34 - 0.95) | 0.42 | (0.15 - 1.15) | 0.76 | (0.45 - 1.31) |
| **Age** | 0.97 | (0.92 - 1.01) | 0.97 | (0.91 - 1.03) | 0.97 | (0.88 - 1.07) | 0.96 | (0.90 - 1.03) |
| **Age Squared** | 1.00* | (1.00 - 1.00) | 1.00 | (1.00 - 1.00) | 1.00 | (1.00 - 1.00) | 1.00 | (1.00 - 1.00) |
| **Education** | 0.95 | (0.91 - 1.00) | 1.02 | (0.95 - 1.09) | 0.99 | (0.87 - 1.12) | 0.99 | (0.92 - 1.06) |
| **Hindu** | 0.65 | (0.41 - 1.03) | 0.56 | (0.29 - 1.11) | 0.63 | (0.12 - 3.30) | 0.48 | (0.22 - 1.04) |
| **Scheduled Caste, Tribe, Other Backwards Class** | 0.85 | (0.52 - 1.38) | 0.88 | (0.50 - 1.55) | 0.74 | (0.12 - 4.71) | 0.95 | (0.48 - 1.87) |
| **Number of Kids** | 1.02 | (0.88 - 1.19) | 1.04 | (0.84 - 1.28) | 0.77 | (0.54 - 1.11) | 1.14 | (0.87 - 1.50) |
| **Household Size** | 1.06 | (0.96 - 1.17) | 0.98 | (0.86 - 1.12) | 1.19 | (0.90 - 1.57) | 0.90 | (0.76 - 1.05) |
| **Poor** | 2.37* | (1.44 - 3.91) | 2.90* | (1.48 - 5.66) | 1.86 | (0.67 - 5.18) | 2.83* | (1.45 - 5.50) |
| **Middle Income** | 0.86 | (0.45 - 1.63) | 1.02 | (0.52 - 2.01) | 0.83 | (0.24 - 2.89) | 0.81 | (0.46 - 1.42) |
|  |  |  |  |  |  |  |  |  |
| **Observations** | 1007 | | 1007 | | 196 | | 811 | |

* p<0.05

** Comparator group is ≥5 Symptoms at Treatment Initiation

|  | **Univariate Regression** | | **Multivariate Regression** | | | | | |
| --- | --- | --- | --- | --- | --- | --- | --- | --- |
|  |  |  | **All Patients** | | **Patients with prior TB** | | **Patients with no prior TB** | |
|  | **OR** | **(95% CI)** | **OR** | **(95% CI)** | **OR** | **(95% CI)** | **OR** | **(95% CI)** |
| **Prior TB Status** |  |  |  |  |  |  |  |  |
| **Prior TB Treatment Episode** | 4.59* | (2.17 - 9.70) | 5.78* | (2.07 - 16.09) |  |  |  |  |
| **Prior TB & completed treatment prior treatment** | 1.63 | (0.58 - 4.62) | 2.08 | (0.67 - 6.45) | 0.72 | (0.22 - 2.33) |  |  |
|  |  |  |  |  |  |  |  |  |
| **Current Illness Treatment and Illness Characteristics** |  |  |  |  |  |  |  |  |
| **Total Delay from Symptom Onset to Treatment Initiation** | 1.01 | (0.97 - 1.05) | 0.96 | (0.91 - 1.02) | 0.96 | (0.86 - 1.06) | 0.96 | (0.89 - 1.03) |
| **Number of Providers Visited** | 2.73* | (1.42 - 5.25) | 2.14 | (0.99 - 4.62) | 4.40* | (1.04 - 18.62) | 1.09 | (0.29 - 4.18) |
| **Treatment or Medication Fees** | 2.79* | (1.16 - 6.71) | 1.26 | (0.44 - 3.58) | 1.40 | (0.14 - 13.76) | 1.71 | (0.46 - 6.39) |
| **Travel Costs** | 2.16* | (1.10 - 4.27) | 1.93 | (0.92 - 4.06) | 0.87 | (0.08 - 9.21) | 1.80 | (0.84 - 3.88) |
| **Treatment, Medication and Travel Costs** | 0.13* | (0.05 - 0.33) | 0.15* | (0.05 - 0.45) | 1.16 | (0.07 - 19.19) | 0.10* | (0.02 - 0.38) |
| **2 or Fewer Symptoms at Treatment Initiation**** | 0.95 | (0.57 - 1.58) | 1.17 | (0.67 - 2.05) | 0.88 | (0.29 - 2.60) | 1.81 | (0.87 - 3.76) |
| **3-4 Symptoms at Treatment Initiation**** | 0.57 | (0.28 - 1.16) | 0.53 | (0.20 - 1.44) | 0.39 | (0.11 - 1.37) | 1.02 | (0.33 - 3.11) |
|  |  |  |  |  |  |  |  |  |
| **Number of Weeks in Care** | 0.96 | (0.90 - 1.03) | 1.04* | (1.01 - 1.07) | 1.07* | (1.02 - 1.13) | 1.01 | (0.98 - 1.05) |
|  |  |  |  |  |  |  |  |  |
| **Patient and Household Characteristics** |  |  |  |  |  |  |  |  |
| **Male** | 0.66* | (0.45 - 0.96) | 0.58* | (0.35 - 0.96) | 0.43 | (0.16 - 1.16) | 0.80 | (0.47 - 1.33) |
| **Age** | 0.97 | (0.92 - 1.01) | 0.97 | (0.91 - 1.03) | 0.97 | (0.88 - 1.07) | 0.96 | (0.90 - 1.02) |
| **Age Squared** | 1.00* | (1.00 - 1.00) | 1.00 | (1.00 - 1.00) | 1.00 | (1.00 - 1.00) | 1.00 | (1.00 - 1.00) |
| **Education** | 0.95 | (0.91 - 1.00) | 1.01 | (0.94 - 1.08) | 0.98 | (0.85 - 1.12) | 0.98 | (0.92 - 1.06) |
| **Hindu** | 0.65 | (0.41 - 1.03) | 0.56 | (0.29 - 1.08) | 0.47 | (0.07 - 3.27) | 0.49 | (0.22 - 1.06) |
| **Scheduled Caste, Tribe, Other Backwards Class** | 0.85 | (0.52 - 1.38) | 0.85 | (0.48 - 1.52) | 0.79 | (0.13 - 4.74) | 0.94 | (0.47 - 1.88) |
| **Number of Kids** | 1.02 | (0.88 - 1.19) | 1.03 | (0.83 - 1.27) | 0.76 | (0.52 - 1.11) | 1.15 | (0.89 - 1.49) |
| **Household Size** | 1.06 | (0.96 - 1.17) | 0.99 | (0.87 - 1.13) | 1.23 | (0.94 - 1.61) | 0.89 | (0.76 - 1.04) |
| **Poor** | 2.37* | (1.44 - 3.91) | 2.93* | (1.50 - 5.69) | 2.00 | (0.66 - 6.05) | 2.79* | (1.47 - 5.30) |
| **Middle Income** | 0.86 | (0.45 - 1.63) | 1.00 | (0.51 - 1.97) | 0.83 | (0.21 - 3.26) | 0.78 | (0.44 - 1.38) |
|  |  |  |  |  |  |  |  |  |
| **Observations** | 1007 | | 1007 | | 196 | | 811 | |

* p<0.05

** Comparator group is ≥5 Symptoms at Treatment Initiation

|  | **Univariate Regression** | | **Multivariate Regression** | | | | | |
| --- | --- | --- | --- | --- | --- | --- | --- | --- |
|  |  |  | **All Patients** | | **Patients with prior TB** | | **Patients with no prior TB** | |
|  | **OR** | **(95% CI)** | **OR** | **(95% CI)** | **OR** | **(95% CI)** | **OR** | **(95% CI)** |
| **Prior TB Status** |  |  |  |  |  |  |  |  |
| **Prior TB Treatment Episode** | 4.59* | (2.17 - 9.70) | 5.57* | (2.01 - 15.41) |  |  |  |  |
| **Prior TB & completed treatment prior treatment** | 1.63 | (0.58 - 4.62) | 2.16 | (0.69 - 6.70) | 0.74 | (0.23 - 2.39) |  |  |
|  |  |  |  |  |  |  |  |  |
| **Current Illness Treatment and Illness Characteristics** |  |  |  |  |  |  |  |  |
| **Total Delay from Symptom Onset to Treatment Initiation** | 1.01 | (0.97 - 1.05) | 0.96 | (0.91 - 1.02) | 0.96 | (0.87 - 1.06) | 0.96 | (0.89 - 1.03) |
| **Number of Providers Visited** | 2.73* | (1.42 - 5.25) | 1.98 | (0.90 - 4.34) | 4.25 | (0.98 - 18.47) | 0.81 | (0.18 - 3.61) |
| **Treatment or Medication Fees** | 2.79* | (1.16 - 6.71) | 1.23 | (0.43 - 3.51) | 1.44 | (0.15 - 13.95) | 1.65 | (0.44 - 6.22) |
| **Travel Costs** | 2.16* | (1.10 - 4.27) | 1.90 | (0.91 - 3.97) | 0.91 | (0.08 - 9.90) | 1.72 | (0.80 - 3.71) |
| **Treatment, Medication and Travel Costs** | 0.13* | (0.05 - 0.33) | 0.15* | (0.05 - 0.45) | 1.13 | (0.07 - 19.30) | 0.09* | (0.02 - 0.38) |
| **2 or Fewer Symptoms at Treatment Initiation**** | 0.95 | (0.57 - 1.58) | 1.17 | (0.67 - 2.05) | 0.94 | (0.29 - 2.99) | 1.79 | (0.86 - 3.72) |
| **3-4 Symptoms at Treatment Initiation**** | 0.57 | (0.28 - 1.16) | 0.53 | (0.19 - 1.45) | 0.40 | (0.11 - 1.43) | 1.03 | (0.33 - 3.17) |
|  |  |  |  |  |  |  |  |  |
| **Number of Weeks in Care** | 0.96 | (0.90 - 1.03) | 1.00 | (0.93 - 1.08) | 1.01 | (0.85 - 1.19) | 0.94 | (0.86 - 1.02) |
| **Number of Weeks in Care ^ 2** | 1.00 | (1.00 - 1.00) | 1.00 | (1.00 - 1.00) | 1.00 | (1.00 - 1.01) | 1.00 | (1.00 - 1.00) |
|  |  |  |  |  |  |  |  |  |
| **Patient and Household Characteristics** |  |  |  |  |  |  |  |  |
| **Male** | 0.66* | (0.45 - 0.96) | 0.58* | (0.35 - 0.96) | 0.43 | (0.16 - 1.17) | 0.80 | (0.48 - 1.33) |
| **Age** | 0.97 | (0.92 - 1.01) | 0.97 | (0.91 - 1.03) | 0.98 | (0.89 - 1.08) | 0.96 | (0.90 - 1.03) |
| **Age Squared** | 1.00* | (1.00 - 1.00) | 1.00 | (1.00 - 1.00) | 1.00 | (1.00 - 1.00) | 1.00 | (1.00 - 1.00) |
| **Education** | 0.95 | (0.91 - 1.00) | 1.01 | (0.94 - 1.08) | 0.97 | (0.84 - 1.12) | 0.98 | (0.92 - 1.05) |
| **Hindu** | 0.65 | (0.41 - 1.03) | 0.58 | (0.30 - 1.15) | 0.50 | (0.08 - 3.28) | 0.53 | (0.25 - 1.15) |
| **Scheduled Caste, Tribe, Other Backwards Class** | 0.85 | (0.52 - 1.38) | 0.83 | (0.46 - 1.49) | 0.71 | (0.11 - 4.55) | 0.92 | (0.47 - 1.83) |
| **Number of Kids** | 1.02 | (0.88 - 1.19) | 1.03 | (0.83 - 1.27) | 0.78 | (0.53 - 1.14) | 1.13 | (0.87 - 1.46) |
| **Household Size** | 1.06 | (0.96 - 1.17) | 0.99 | (0.87 - 1.13) | 1.21 | (0.92 - 1.60) | 0.90 | (0.77 - 1.06) |
| **Poor** | 2.37* | (1.44 - 3.91) | 2.93* | (1.50 - 5.73) | 1.99 | (0.66 - 6.00) | 2.75* | (1.45 - 5.21) |
| **Middle Income** | 0.86 | (0.45 - 1.63) | 1.00 | (0.51 - 1.99) | 0.87 | (0.22 - 3.46) | 0.78 | (0.44 - 1.38) |
|  |  |  |  |  |  |  |  |  |
| **Observations** | 1007 | | 1007 | | 196 | | 811 | |

* p<0.05

** Comparator group is ≥5 Symptoms at Treatment Initiation
