# Supplementary material for: Tuberculosis treatment discontinuation and symptom persistence: an observational study of Bihar, India’s public care system covering >100,000,000 inhabitants
Source: BMC Public Health. 2014 May 1;14:418. doi: 10.1186/1471-2458-14-418 (PMC4041057; doi:10.1186/1471-2458-14-418)
Supplement: Additional file 5: Table S5 — Negative Binomial Model of Number of Symptoms Presenting 25 Weeks After Initiating Treatment. [file 1471-2458-14-418-S5.docx]

**Additional file 5: Table S5: Negative Binomial Model of Number of Symptoms Presenting 25 Weeks After Initiating Treatment**

|  | **Univariate Regression** | | **Multivariate Regression** | | | | | |
| --- | --- | --- | --- | --- | --- | --- | --- | --- |
|  |  |  | **All Patients** | | **Patients with prior TB** | | **Patients with no prior TB** | |
|  | **IRR** | **(95% CI)** | **IRR** | **(95% CI)** | **IRR** | **(95% CI)** | **IRR** | **(95% CI)** |
| **Prior TB Status** |  |  |  |  |  |  |  |  |
| **Prior TB Treatment Episode** | 0.90* | (0.50 - 1.30) | 0.94* | (0.34 - 1.55) |  |  |  |  |
| **Prior TB & completed treatment prior treatment** | 0.58* | (0.14 - 1.01) | 0.54* | (0.02 - 1.06) | -0.24 | (-1.14 - 0.66) |  |  |
|  |  |  |  |  |  |  |  |  |
| **Current Illness Treatment and Illness Characteristics** |  |  |  |  |  |  |  |  |
| **Total Delay from Symptom Onset to Treatment Initiation** | -0.00 | (-0.03 - 0.03) | -0.01 | (-0.04 - 0.01) | -0.05 | (-0.11 - 0.01) | -0.00 | (-0.01 - 0.01) |
| **Number of Providers Visited** | 0.54* | (0.22 - 0.86) | 0.07 | (-0.17 - 0.31) | 0.59 | (-0.01 - 1.18) | 0.08 | (-0.19 - 0.34) |
| **Treatment or Medication Fees** | 0.78* | (0.40 - 1.15) | 0.18 | (-0.15 - 0.51) | -0.02 | (-1.09 - 1.04) | 0.21* | (0.01 - 0.41) |
| **Travel Costs** | 0.35* | (0.05 - 0.66) | 0.28* | (0.08 - 0.48) | -0.82 | (-2.63 - 1.00) | 0.14* | (0.04 - 0.25) |
| **Treatment, Medication and Travel Costs** | -1.30* | (-1.91 - -0.68) | -0.95* | (-1.46 - -0.44) | 0.69 | (-0.84 - 2.22) | -0.77* | (-1.23 - -0.31) |
| **2 or Fewer Symptoms at Treatment Initiation**** | -0.32* | (-0.63 - -0.01) | -0.02 | (-0.21 - 0.18) | -0.13 | (-0.90 - 0.63) | 0.13 | (-0.02 - 0.27) |
| **3-4 Symptoms at Treatment Initiation**** | -0.42* | (-0.70 - -0.15) | -0.21 | (-0.46 - 0.05) | -0.75 | (-1.55 - 0.05) | 0.05 | (-0.15 - 0.26) |
|  |  |  |  |  |  |  |  |  |
| **Patient and Household Characteristics** |  |  |  |  |  |  |  |  |
| **Male** | -0.27 | (-0.56 - 0.02) | -0.17 | (-0.36 - 0.02) | -0.47 | (-1.15 - 0.22) | -0.03 | (-0.14 - 0.08) |
| **Age** | -0.02* | (-0.05 - -0.00) | -0.01 | (-0.03 - 0.01) | 0.00 | (-0.05 - 0.06) | -0.01 | (-0.02 - 0.00) |
| **Age Squared** | 0.00* | (0.00 - 0.00) | 0.00 | (-0.00 - 0.00) | 0.00 | (-0.00 - 0.00) | 0.00 | (-0.00 - 0.00) |
| **Education** | -0.05* | (-0.08 - -0.02) | -0.01 | (-0.03 - 0.02) | -0.03 | (-0.10 - 0.04) | -0.01 | (-0.02 - 0.01) |
| **Hindu** | -0.24 | (-0.60 - 0.13) | -0.19 | (-0.45 - 0.08) | -0.09 | (-0.91 - 0.74) | -0.16 | (-0.38 - 0.06) |
| **Scheduled Caste, Tribe, Other Backwards Class** | 0.05 | (-0.26 - 0.36) | -0.03 | (-0.25 - 0.19) | 0.21 | (-0.51 - 0.93) | 0.01 | (-0.13 - 0.15) |
| **Number of Kids** | 0.04 | (-0.05 - 0.12) | 0.04 | (-0.03 - 0.11) | -0.13 | (-0.33 - 0.07) | 0.04 | (-0.01 - 0.09) |
| **Household Size** | 0.04 | (-0.00 - 0.09) | -0.00 | (-0.05 - 0.04) | 0.05 | (-0.09 - 0.20) | -0.02 | (-0.05 - 0.01) |
| **Poor** | 0.50* | (0.26 - 0.75) | 0.38* | (0.15 - 0.61) | 0.42 | (-0.28 - 1.12) | 0.22* | (0.08 - 0.36) |
| **Middle Income** | -0.04 | (-0.34 - 0.25) | 0.08 | (-0.16 - 0.33) | -0.10 | (-0.87 - 0.68) | 0.02 | (-0.10 - 0.14) |
|  |  |  |  |  |  |  |  |  |
| **Observations** | 1007 | | 1007 | | 196 | | 811 | |

* p<0.05

** Comparator group is ≥5 Symptoms at Treatment Initiation

|  | **Univariate Regression** | | **Multivariate Regression** | | | | | |
| --- | --- | --- | --- | --- | --- | --- | --- | --- |
|  |  |  | **All Patients** | | **Patients with prior TB** | | **Patients with no prior TB** | |
|  | **IRR** | **(95% CI)** | **IRR** | **(95% CI)** | **IRR** | **(95% CI)** | **IRR** | **(95% CI)** |
| **Prior TB Status** |  |  |  |  |  |  |  |  |
| **Prior TB Treatment Episode** | 0.90* | (0.50 - 1.30) | 0.93* | (0.31 - 1.54) |  |  |  |  |
| **Prior TB & completed treatment prior treatment** | 0.58* | (0.14 - 1.01) | 0.52 | (-0.05 - 1.09) | -0.35 | (-1.29 - 0.58) |  |  |
|  |  |  |  |  |  |  |  |  |
| **Current Illness Treatment and Illness Characteristics** |  |  |  |  |  |  |  |  |
| **Total Delay from Symptom Onset to Treatment Initiation** | -0.00 | (-0.03 - 0.03) | -0.02 | (-0.04 - 0.00) | -0.04 | (-0.10 - 0.02) | -0.00 | (-0.01 - 0.01) |
| **Number of Providers Visited** | 0.54* | (0.22 - 0.86) | 0.11 | (-0.14 - 0.36) | 0.71* | (0.05 - 1.37) | 0.06 | (-0.18 - 0.30) |
| **Treatment or Medication Fees** | 0.78* | (0.40 - 1.15) | 0.15 | (-0.17 - 0.46) | -0.05 | (-1.24 - 1.14) | 0.17 | (-0.02 - 0.37) |
| **Travel Costs** | 0.35* | (0.05 - 0.66) | 0.24* | (0.05 - 0.43) | -1.09 | (-3.24 - 1.06) | 0.11* | (0.02 - 0.20) |
| **Treatment, Medication and Travel Costs** | -1.30* | (-1.91 - -0.68) | -0.84* | (-1.33 - -0.36) | 1.11 | (-0.63 - 2.84) | -0.65* | (-1.07 - -0.24) |
| **2 or Fewer Symptoms at Treatment Initiation**** | -0.32* | (-0.63 - -0.01) | 0.00 | (-0.19 - 0.19) | 0.08 | (-0.76 - 0.93) | 0.13* | (0.00 - 0.26) |
| **3-4 Symptoms at Treatment Initiation**** | -0.42* | (-0.70 - -0.15) | -0.23 | (-0.47 - 0.01) | -0.77 | (-1.59 - 0.04) | 0.04 | (-0.15 - 0.22) |
|  |  |  |  |  |  |  |  |  |
| **Treated < 8 Weeks** | 0.50* | (0.12 - 0.88) | -0.04 | (-0.31 - 0.22) | -0.07 | (-0.95 - 0.82) | 0.01 | (-0.24 - 0.26) |
| **Treated 9-16 Weeks** | 0.01 | (-0.47 - 0.49) | 0.00 | (-0.37 - 0.37) | -0.82 | (-2.05 - 0.41) | 0.10 | (-0.06 - 0.26) |
|  |  |  |  |  |  |  |  |  |
| **Patient and Household Characteristics** |  |  |  |  |  |  |  |  |
| **Male** | -0.27 | (-0.56 - 0.02) | -0.15 | (-0.33 - 0.03) | -0.49 | (-1.20 - 0.21) | -0.01 | (-0.11 - 0.09) |
| **Age** | -0.02* | (-0.05 - -0.00) | -0.01 | (-0.03 - 0.01) | -0.01 | (-0.07 - 0.06) | -0.01 | (-0.02 - 0.00) |
| **Age Squared** | 0.00* | (0.00 - 0.00) | 0.00 | (-0.00 - 0.00) | 0.00 | (-0.00 - 0.00) | 0.00 | (-0.00 - 0.00) |
| **Education** | -0.05* | (-0.08 - -0.02) | -0.01 | (-0.03 - 0.01) | -0.04 | (-0.12 - 0.04) | -0.01 | (-0.02 - 0.01) |
| **Hindu** | -0.24 | (-0.60 - 0.13) | -0.18 | (-0.44 - 0.09) | -0.07 | (-1.03 - 0.89) | -0.14 | (-0.35 - 0.07) |
| **Scheduled Caste, Tribe, Other Backwards Class** | 0.05 | (-0.26 - 0.36) | -0.02 | (-0.23 - 0.19) | 0.07 | (-0.81 - 0.95) | 0.01 | (-0.13 - 0.14) |
| **Number of Kids** | 0.04 | (-0.05 - 0.12) | 0.05 | (-0.02 - 0.12) | -0.13 | (-0.34 - 0.09) | 0.05 | (-0.00 - 0.09) |
| **Household Size** | 0.04 | (-0.00 - 0.09) | -0.01 | (-0.05 - 0.04) | 0.03 | (-0.13 - 0.19) | -0.02 | (-0.05 - 0.01) |
| **Poor** | 0.50* | (0.26 - 0.75) | 0.35* | (0.13 - 0.56) | 0.33 | (-0.43 - 1.10) | 0.19* | (0.06 - 0.31) |
| **Middle Income** | -0.04 | (-0.34 - 0.25) | 0.06 | (-0.17 - 0.28) | -0.11 | (-0.93 - 0.70) | 0.01 | (-0.10 - 0.12) |
|  |  |  |  |  |  |  |  |  |
| **Observations** | 1007 | | 1007 | | 196 | | 811 | |

* p<0.05

** Comparator group is ≥5 Symptoms at Treatment Initiation

|  | **Univariate Regression** | | **Multivariate Regression** | | | | | |
| --- | --- | --- | --- | --- | --- | --- | --- | --- |
|  |  |  | **All Patients** | | **Patients with prior TB** | | **Patients with no prior TB** | |
|  | **IRR** | **(95% CI)** | **IRR** | **(95% CI)** | **IRR** | **(95% CI)** | **IRR** | **(95% CI)** |
| **Prior TB Status** |  |  |  |  |  |  |  |  |
| **Prior TB Treatment Episode** | 0.90* | (0.50 - 1.30) | 1.01* | (0.36 - 1.65) |  |  |  |  |
| **Prior TB & completed treatment prior treatment** | 0.58* | (0.14 - 1.01) | 0.45 | (-0.07 - 0.97) | -0.35 | (-1.31 - 0.61) |  |  |
|  |  |  |  |  |  |  |  |  |
| **Current Illness Treatment and Illness Characteristics** |  |  |  |  |  |  |  |  |
| **Total Delay from Symptom Onset to Treatment Initiation** | -0.00 | (-0.03 - 0.03) | -0.02 | (-0.04 - 0.00) | -0.05 | (-0.11 - 0.01) | -0.00 | (-0.01 - 0.01) |
| **Number of Providers Visited** | 0.54* | (0.22 - 0.86) | 0.12 | (-0.12 - 0.36) | 0.71* | (0.03 - 1.38) | 0.06 | (-0.19 - 0.31) |
| **Treatment or Medication Fees** | 0.78* | (0.40 - 1.15) | 0.16 | (-0.15 - 0.48) | -0.09 | (-1.28 - 1.10) | 0.17 | (-0.02 - 0.36) |
| **Travel Costs** | 0.35* | (0.05 - 0.66) | 0.24* | (0.06 - 0.43) | -0.96 | (-2.98 - 1.06) | 0.11* | (0.01 - 0.20) |
| **Treatment, Medication and Travel Costs** | -1.30* | (-1.91 - -0.68) | -0.85* | (-1.34 - -0.36) | 0.94 | (-0.72 - 2.61) | -0.63* | (-1.04 - -0.23) |
| **2 or Fewer Symptoms at Treatment Initiation**** | -0.32* | (-0.63 - -0.01) | 0.01 | (-0.19 - 0.20) | 0.00 | (-0.81 - 0.81) | 0.12 | (-0.01 - 0.25) |
| **3-4 Symptoms at Treatment Initiation**** | -0.42* | (-0.70 - -0.15) | -0.23 | (-0.47 - 0.01) | -0.82 | (-1.67 - 0.03) | 0.02 | (-0.16 - 0.21) |
|  |  |  |  |  |  |  |  |  |
| **Completed At Least 25 Weeks of Treatment** | -0.09 | (-0.38 - 0.21) | 0.08 | (-0.14 - 0.31) | 0.25 | (-0.58 - 1.08) | -0.04 | (-0.15 - 0.07) |
|  |  |  |  |  |  |  |  |  |
| **Patient and Household Characteristics** |  |  |  |  |  |  |  |  |
| **Male** | -0.27 | (-0.56 - 0.02) | -0.15 | (-0.33 - 0.03) | -0.51 | (-1.23 - 0.21) | -0.01 | (-0.10 - 0.08) |
| **Age** | -0.02* | (-0.05 - -0.00) | -0.01 | (-0.03 - 0.01) | -0.01 | (-0.08 - 0.06) | -0.01 | (-0.02 - 0.00) |
| **Age Squared** | 0.00* | (0.00 - 0.00) | 0.00 | (-0.00 - 0.00) | 0.00 | (-0.00 - 0.00) | 0.00 | (-0.00 - 0.00) |
| **Education** | -0.05* | (-0.08 - -0.02) | -0.01 | (-0.03 - 0.01) | -0.04 | (-0.12 - 0.04) | -0.01 | (-0.02 - 0.01) |
| **Hindu** | -0.24 | (-0.60 - 0.13) | -0.18 | (-0.44 - 0.07) | -0.08 | (-1.03 - 0.86) | -0.14 | (-0.34 - 0.06) |
| **Scheduled Caste, Tribe, Other Backwards Class** | 0.05 | (-0.26 - 0.36) | -0.02 | (-0.23 - 0.18) | 0.27 | (-0.49 - 1.04) | 0.01 | (-0.13 - 0.14) |
| **Number of Kids** | 0.04 | (-0.05 - 0.12) | 0.05 | (-0.02 - 0.11) | -0.12 | (-0.33 - 0.09) | 0.04 | (-0.00 - 0.09) |
| **Household Size** | 0.04 | (-0.00 - 0.09) | -0.01 | (-0.05 - 0.04) | 0.04 | (-0.12 - 0.21) | -0.02 | (-0.05 - 0.01) |
| **Poor** | 0.50* | (0.26 - 0.75) | 0.35* | (0.13 - 0.56) | 0.35 | (-0.42 - 1.12) | 0.18* | (0.06 - 0.30) |
| **Middle Income** | -0.04 | (-0.34 - 0.25) | 0.05 | (-0.17 - 0.28) | -0.13 | (-0.97 - 0.70) | 0.00 | (-0.11 - 0.11) |
|  |  |  |  |  |  |  |  |  |
| **Observations** | 1007 | | 1007 | | 196 | | 811 | |

* p<0.05

** Comparator group is ≥5 Symptoms at Treatment Initiation

|  | **Univariate Regression** | | **Multivariate Regression** | | | | | |
| --- | --- | --- | --- | --- | --- | --- | --- | --- |
|  |  |  | **All Patients** | | **Patients with prior TB** | | **Patients with no prior TB** | |
|  | **IRR** | **(95% CI)** | **IRR** | **(95% CI)** | **IRR** | **(95% CI)** | **IRR** | **(95% CI)** |
| **Prior TB Status** |  |  |  |  |  |  |  |  |
| **Prior TB Treatment Episode** | 0.90* | (0.50 - 1.30) | 1.06* | (0.39 - 1.74) |  |  |  |  |
| **Prior TB & completed treatment prior treatment** | 0.58* | (0.14 - 1.01) | 0.40 | (-0.09 - 0.88) | -0.40 | (-1.34 - 0.54) |  |  |
|  |  |  |  |  |  |  |  |  |
| **Current Illness Treatment and Illness Characteristics** |  |  |  |  |  |  |  |  |
| **Total Delay from Symptom Onset to Treatment Initiation** | -0.00 | (-0.03 - 0.03) | -0.02 | (-0.04 - 0.00) | -0.04 | (-0.10 - 0.02) | -0.00 | (-0.01 - 0.01) |
| **Number of Providers Visited** | 0.54* | (0.22 - 0.86) | 0.14 | (-0.09 - 0.38) | 0.76* | (0.09 - 1.43) | 0.08 | (-0.16 - 0.32) |
| **Treatment or Medication Fees** | 0.78* | (0.40 - 1.15) | 0.16 | (-0.16 - 0.48) | -0.07 | (-1.26 - 1.12) | 0.18 | (-0.01 - 0.37) |
| **Travel Costs** | 0.35* | (0.05 - 0.66) | 0.24* | (0.06 - 0.43) | -1.00 | (-3.01 - 1.01) | 0.11* | (0.02 - 0.20) |
| **Treatment, Medication and Travel Costs** | -1.30* | (-1.91 - -0.68) | -0.86* | (-1.36 - -0.36) | 0.96 | (-0.71 - 2.62) | -0.65* | (-1.07 - -0.23) |
| **2 or Fewer Symptoms at Treatment Initiation**** | -0.32* | (-0.63 - -0.01) | 0.01 | (-0.19 - 0.20) | 0.00 | (-0.81 - 0.81) | 0.12 | (-0.01 - 0.25) |
| **3-4 Symptoms at Treatment Initiation**** | -0.42* | (-0.70 - -0.15) | -0.23 | (-0.47 - 0.01) | -0.84 | (-1.68 - 0.01) | 0.02 | (-0.16 - 0.20) |
|  |  |  |  |  |  |  |  |  |
| **Number of Weeks in Care** | -0.04* | (-0.07 - -0.01) | 0.01 | (-0.00 - 0.02) | 0.02 | (-0.01 - 0.05) | 0.00 | (-0.00 - 0.01) |
|  |  |  |  |  |  |  |  |  |
| **Patient and Household Characteristics** |  |  |  |  |  |  |  |  |
| **Male** | -0.27 | (-0.56 - 0.02) | -0.14 | (-0.32 - 0.03) | -0.49 | (-1.21 - 0.22) | -0.01 | (-0.10 - 0.08) |
| **Age** | -0.02* | (-0.05 - -0.00) | -0.01 | (-0.03 - 0.01) | -0.01 | (-0.08 - 0.06) | -0.01 | (-0.02 - 0.00) |
| **Age Squared** | 0.00* | (0.00 - 0.00) | 0.00 | (-0.00 - 0.00) | 0.00 | (-0.00 - 0.00) | 0.00 | (-0.00 - 0.00) |
| **Education** | -0.05* | (-0.08 - -0.02) | -0.01 | (-0.03 - 0.01) | -0.05 | (-0.12 - 0.03) | -0.01 | (-0.02 - 0.01) |
| **Hindu** | -0.24 | (-0.60 - 0.13) | -0.20 | (-0.45 - 0.05) | -0.20 | (-1.26 - 0.85) | -0.14 | (-0.34 - 0.06) |
| **Scheduled Caste, Tribe, Other Backwards Class** | 0.05 | (-0.26 - 0.36) | -0.03 | (-0.24 - 0.18) | 0.27 | (-0.50 - 1.04) | 0.01 | (-0.12 - 0.14) |
| **Number of Kids** | 0.04 | (-0.05 - 0.12) | 0.04 | (-0.02 - 0.11) | -0.12 | (-0.33 - 0.09) | 0.04 | (-0.00 - 0.09) |
| **Household Size** | 0.04 | (-0.00 - 0.09) | -0.01 | (-0.05 - 0.04) | 0.05 | (-0.11 - 0.22) | -0.02 | (-0.05 - 0.01) |
| **Poor** | 0.50* | (0.26 - 0.75) | 0.35* | (0.13 - 0.56) | 0.39 | (-0.36 - 1.14) | 0.18* | (0.07 - 0.30) |
| **Middle Income** | -0.04 | (-0.34 - 0.25) | 0.05 | (-0.17 - 0.28) | -0.14 | (-0.94 - 0.67) | -0.00 | (-0.11 - 0.10) |
|  |  |  |  |  |  |  |  |  |
| **Observations** | 1007 | | 1007 | | 196 | | 811 | |

* p<0.05

** Comparator group is ≥5 Symptoms at Treatment Initiation

|  | **Univariate Regression** | | **Multivariate Regression** | | | | | |
| --- | --- | --- | --- | --- | --- | --- | --- | --- |
|  |  |  | **All Patients** | | **Patients with prior TB** | | **Patients with no prior TB** | |
|  | **IRR** | **(95% CI)** | **IRR** | **(95% CI)** | **IRR** | **(95% CI)** | **IRR** | **(95% CI)** |
| **Prior TB Status** |  |  |  |  |  |  |  |  |
| **Prior TB Treatment Episode** | 0.90* | (0.50 - 1.30) | 1.01* | (0.37 - 1.65) |  |  |  |  |
| **Prior TB & completed treatment prior treatment** | 0.58* | (0.14 - 1.01) | 0.43 | (-0.07 - 0.93) | -0.38 | (-1.32 - 0.55) |  |  |
|  |  |  |  |  |  |  |  |  |
| **Current Illness Treatment and Illness Characteristics** |  |  |  |  |  |  |  |  |
| **Total Delay from Symptom Onset to Treatment Initiation** | -0.00 | (-0.03 - 0.03) | -0.02 | (-0.04 - 0.00) | -0.04 | (-0.10 - 0.03) | -0.00 | (-0.01 - 0.01) |
| **Number of Providers Visited** | 0.54* | (0.22 - 0.86) | 0.12 | (-0.13 - 0.37) | 0.70* | (0.02 - 1.38) | 0.05 | (-0.21 - 0.32) |
| **Treatment or Medication Fees** | 0.78* | (0.40 - 1.15) | 0.16 | (-0.16 - 0.48) | 0.01 | (-1.13 - 1.14) | 0.18 | (-0.02 - 0.37) |
| **Travel Costs** | 0.35* | (0.05 - 0.66) | 0.24* | (0.06 - 0.43) | -0.85 | (-2.78 - 1.08) | 0.11* | (0.01 - 0.20) |
| **Treatment, Medication and Travel Costs** | -1.30* | (-1.91 - -0.68) | -0.87* | (-1.38 - -0.37) | 0.86 | (-0.76 - 2.48) | -0.68* | (-1.11 - -0.25) |
| **2 or Fewer Symptoms at Treatment Initiation**** | -0.32* | (-0.63 - -0.01) | 0.01 | (-0.18 - 0.21) | 0.05 | (-0.77 - 0.88) | 0.12 | (-0.02 - 0.25) |
| **3-4 Symptoms at Treatment Initiation**** | -0.42* | (-0.70 - -0.15) | -0.22 | (-0.46 - 0.02) | -0.81 | (-1.66 - 0.05) | 0.02 | (-0.17 - 0.21) |
|  |  |  |  |  |  |  |  |  |
| **Number of Weeks in Care** | -0.04* | (-0.07 - -0.01) | -0.01 | (-0.03 - 0.02) | -0.00 | (-0.07 - 0.06) | -0.01 | (-0.03 - 0.00) |
| **Number of Weeks in Care ^ 2** | 0.00* | (0.00 - 0.00) | 0.00 | (-0.00 - 0.00) | 0.00 | (-0.00 - 0.00) | 0.00 | (-0.00 - 0.00) |
|  |  |  |  |  |  |  |  |  |
| **Patient and Household Characteristics** |  |  |  |  |  |  |  |  |
| **Male** | -0.27 | (-0.56 - 0.02) | -0.14 | (-0.31 - 0.04) | -0.50 | (-1.22 - 0.22) | 0.00 | (-0.09 - 0.09) |
| **Age** | -0.02* | (-0.05 - -0.00) | -0.01 | (-0.03 - 0.01) | -0.01 | (-0.08 - 0.05) | -0.01 | (-0.02 - 0.00) |
| **Age Squared** | 0.00* | (0.00 - 0.00) | 0.00 | (-0.00 - 0.00) | 0.00 | (-0.00 - 0.00) | 0.00 | (-0.00 - 0.00) |
| **Education** | -0.05* | (-0.08 - -0.02) | -0.01 | (-0.03 - 0.01) | -0.06 | (-0.14 - 0.02) | -0.01 | (-0.02 - 0.00) |
| **Hindu** | -0.24 | (-0.60 - 0.13) | -0.17 | (-0.42 - 0.08) | -0.17 | (-1.23 - 0.88) | -0.12 | (-0.31 - 0.07) |
| **Scheduled Caste, Tribe, Other Backwards Class** | 0.05 | (-0.26 - 0.36) | -0.04 | (-0.25 - 0.17) | 0.23 | (-0.54 - 1.00) | 0.00 | (-0.14 - 0.14) |
| **Number of Kids** | 0.04 | (-0.05 - 0.12) | 0.04 | (-0.03 - 0.11) | -0.11 | (-0.32 - 0.09) | 0.04 | (-0.01 - 0.09) |
| **Household Size** | 0.04 | (-0.00 - 0.09) | -0.00 | (-0.05 - 0.04) | 0.04 | (-0.13 - 0.21) | -0.02 | (-0.05 - 0.01) |
| **Poor** | 0.50* | (0.26 - 0.75) | 0.34* | (0.12 - 0.56) | 0.34 | (-0.46 - 1.14) | 0.18* | (0.06 - 0.30) |
| **Middle Income** | -0.04 | (-0.34 - 0.25) | 0.05 | (-0.18 - 0.29) | -0.18 | (-1.04 - 0.69) | -0.00 | (-0.11 - 0.11) |
|  |  |  |  |  |  |  |  |  |
| **Observations** | 1007 | | 1007 | | 196 | | 811 | |

* p<0.05

** Comparator group is ≥5 Symptoms at Treatment Initiation
