# Supplementary material for: Tuberculosis treatment discontinuation and symptom persistence: an observational study of Bihar, India’s public care system covering >100,000,000 inhabitants
Source: BMC Public Health. 2014 May 1;14:418. doi: 10.1186/1471-2458-14-418 (PMC4041057; doi:10.1186/1471-2458-14-418)
Supplement: Additional file 6: Table S6 — Likelihood of Symptom Persistence 16 Weeks after Treatment Initiation: Logistic Regression Results. [file 1471-2458-14-418-S6.docx]

**Additional file 6: Table S6. Likelihood of Symptom Persistence 16 Weeks after Treatment Initiation: Logistic Regression Results**

|  | **Univariate Regression** | | **Multivariate Regression** | | | | | |
| --- | --- | --- | --- | --- | --- | --- | --- | --- |
|  | **All Patients** | | **All Patients** | | **Patients with prior TB** | | **Patients with no prior TB** | |
|  | **OR** | **(95% CI)** | **OR** | **(95% CI)** | **OR** | **(95% CI)** | **OR** | **(95% CI)** |
|  |  |  |  |  |  |  |  |  |
| **Prior TB Status** | | | | | | | | |
| **Prior TB Treatment Episode** | 4.42* | (2.04 - 9.61) | 4.91* | (1.84 - 13.14) |  |  |  |  |
| **Prior TB & Completed Prior Treatment** | 1.78 | (0.62 - 5.16) | 2.52 | (0.82 - 7.75) | 0.92 | (0.31 - 2.73) |  |  |
|  |  |  |  |  |  |  |  |  |
| **Current Illness Treatment and Illness Characteristics** | | | | | | | | |
| **Total Weeks from Symptom Onset**  **to Treatment Initiation** | 1.01 | (0.96 - 1.05) | 0.96 | (0.91 - 1.01) | 0.94 | (0.85 - 1.04) | 0.96 | (0.89 - 1.03) |
| **Number of Providers Visited** | 2.80* | (1.44 - 5.43) | 2.12 | (0.94 - 4.79) | 4.51* | (1.15 - 17.64) | 1.05 | (0.23 - 4.77) |
| **Treatment or Medication Fees** | 3.00* | (1.26 - 7.19) | 1.30 | (0.47 - 3.58) | 1.74 | (0.16 - 18.54) | 1.83 | (0.49 - 6.80) |
| **Travel Costs** | 2.16* | (1.09 - 4.29) | 1.89 | (0.92 - 3.88) | 0.82 | (0.07 - 9.04) | 1.74 | (0.82 - 3.71) |
| **Treatment, Medication and Travel Costs** | 0.13* | (0.05 - 0.32) | 0.16* | (0.06 - 0.45) | 1.19 | (0.07 - 19.80) | 0.09* | (0.02 - 0.37) |
|  |  |  |  |  |  |  |  |  |
| **Remained in Care 0-8 Weeks** | 1.55 | (0.72 - 3.35) | 1.14 | (0.65 - 1.99) | 0.98 | (0.29 - 3.29) | 2.15* | (1.06 - 4.35) |
| **Remained in Care 9-16 weeks** | 0.84 | (0.27 - 2.58) | 0.50 | (0.17 - 1.47) | 0.39 | (0.11 - 1.39) | 1.22 | (0.41 - 3.64) |
|  |  |  |  |  |  |  |  |  |
| **2 or Fewer Symptoms at Treatment Initiation**** | 0.93 | (0.55 - 1.57) | 1.14 | (0.65 - 1.99) | 0.98 | (0.29 - 3.29) | 2.15* | (1.06 - 4.35) |
| **3-4 Symptoms at Treatment Initiation**** | 0.58 | (0.28 - 1.17) | 0.50 | (0.17 - 1.47) | 0.39 | (0.11 - 1.39) | 1.22 | (0.41 - 3.64) |
|  |  |  |  |  |  |  |  |  |
| **Patient and Household Characteristics** | | | | | | | | |
| **Male** | 0.63* | (0.43 - 0.93) | 0.53* | (0.31 - 0.88) | 0.39 | (0.14 - 1.07) | 0.72 | (0.42 - 1.23) |
| **Age** | 0.96 | (0.92 - 1.01) | 0.97 | (0.91 - 1.03) | 0.96 | (0.87 - 1.07) | 0.96 | (0.89 - 1.03) |
| **Age Squared** | 1.00* | (1.00 - 1.00) | 1.00 | (1.00 - 1.00) | 1.00 | (1.00 - 1.00) | 1.00 | (1.00 - 1.00) |
| **Education** | 0.95 | (0.90 - 1.00) | 1.01 | (0.95 - 1.08) | 1.00 | (0.88 - 1.14) | 0.98 | (0.91 - 1.05) |
| **Hindu** | 0.65 | (0.41 - 1.05) | 0.55 | (0.27 - 1.11) | 0.62 | (0.09 - 4.21) | 0.49 | (0.22 - 1.10) |
| **Scheduled Caste, Tribe, Other Backwards Class** | 0.87 | (0.53 - 1.43) | 0.91 | (0.50 - 1.64) | 0.82 | (0.10 - 6.45) | 0.93 | (0.45 - 1.93) |
| **Number of Kids** | 1.01 | (0.87 - 1.18) | 1.01 | (0.81 - 1.25) | 0.75 | (0.51 - 1.09) | 1.09 | (0.84 - 1.43) |
| **Household Size** | 1.06 | (0.96 - 1.17) | 1.00 | (0.88 - 1.14) | 1.21 | (0.90 - 1.63) | 0.92 | (0.78 - 1.07) |
| **Poor** | 2.36* | (1.42 - 3.92) | 2.90* | (1.46 - 5.75) | 2.14 | (0.72 - 6.36) | 2.85* | (1.41 - 5.74) |
| **Middle Income** | 0.83 | (0.42 - 1.61) | 0.96 | (0.47 - 1.96) | 0.89 | (0.26 - 3.04) | 0.74 | (0.39 - 1.39) |
|  |  |  |  |  |  |  |  |  |
| **Observations** | 1007 | | 1007 | | 196 | | 811 | |

* p<0.05

** Comparator group is ≥5 Symptoms at Treatment Initiation
