# Supplementary figures and images for: Tuberculosis treatment discontinuation and symptom persistence: an observational study of Bihar, India’s public care system covering >100,000,000 inhabitants
Source: BMC Public Health. 2014 May 1;14:418. doi: 10.1186/1471-2458-14-418 (PMC4041057; doi:10.1186/1471-2458-14-418)

**Additional file 7: Share of Patients Ending Treatment by Category II Treatment Phase**


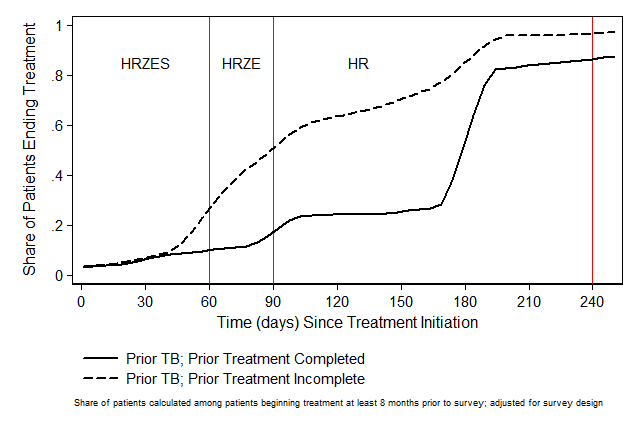

Supplement: Additional file 7 — Share of Patients Ending Treatment by Category II Treatment Phase. [file 1471-2458-14-418-S7.docx]

**Additional file 8: Case Detection Proportions Reported by RNTCP**


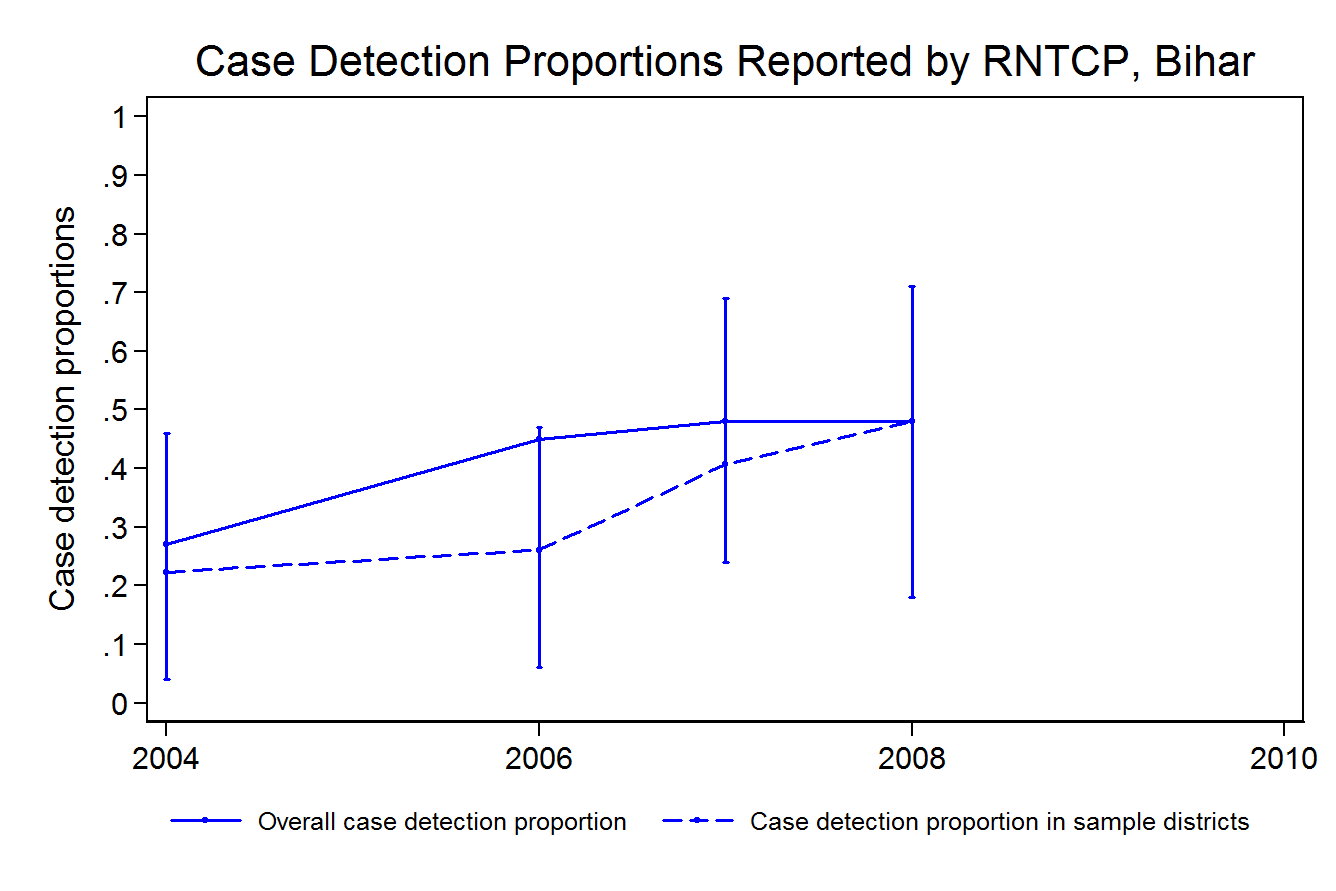

Supplement: Additional file 8 — Case Detection Proportions Reported by RNTCP. [file 1471-2458-14-418-S8.docx]

**Additional file 9: 3 Month Case Conversion Rate for Bihar Reported by RNTCP**


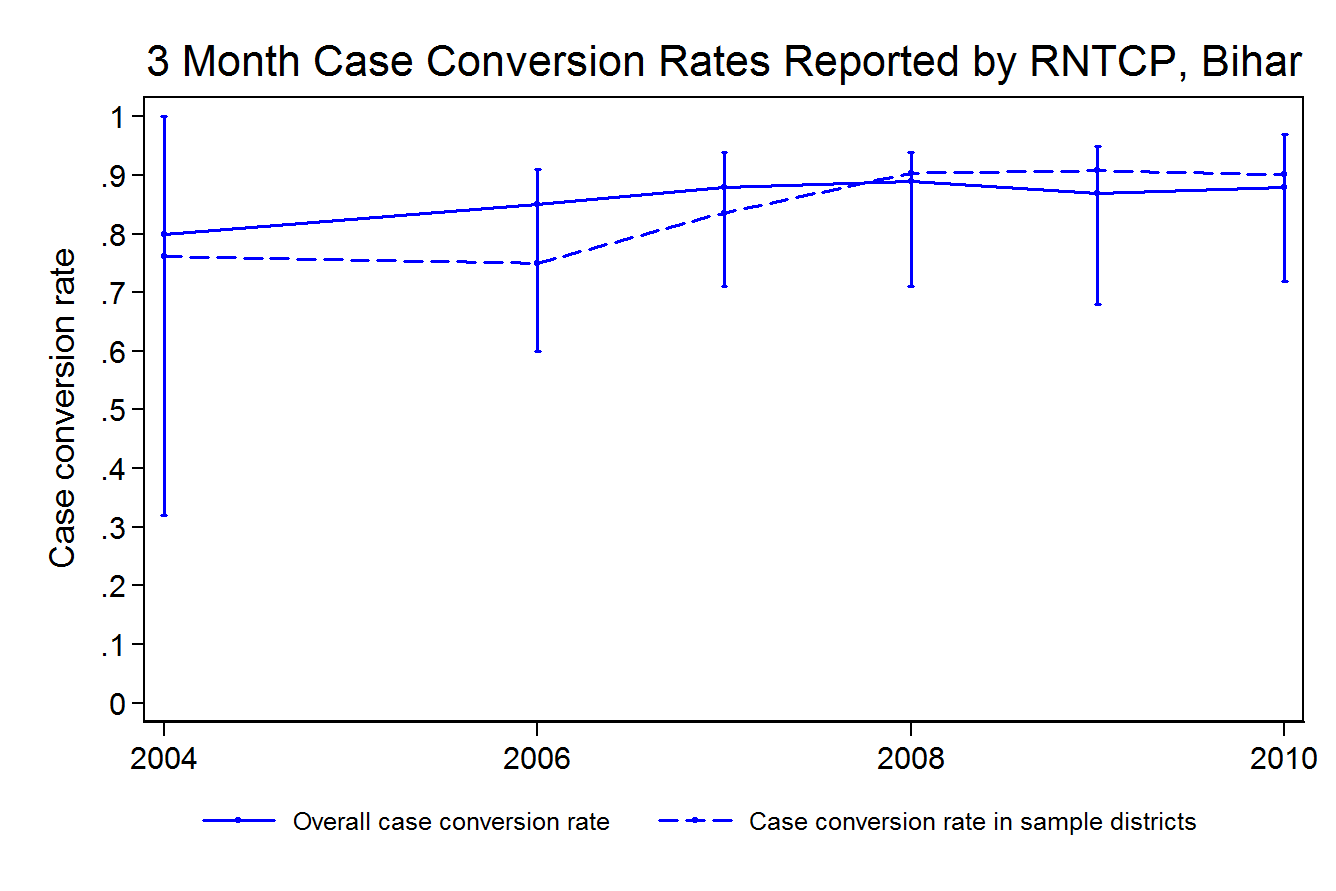

Supplement: Additional file 9 — 3 Month Case Conversion Rate for Bihar Reported by RNTCP. [file 1471-2458-14-418-S9.docx]

**Additional file 10: Cure Rate Reported by RNTCP**


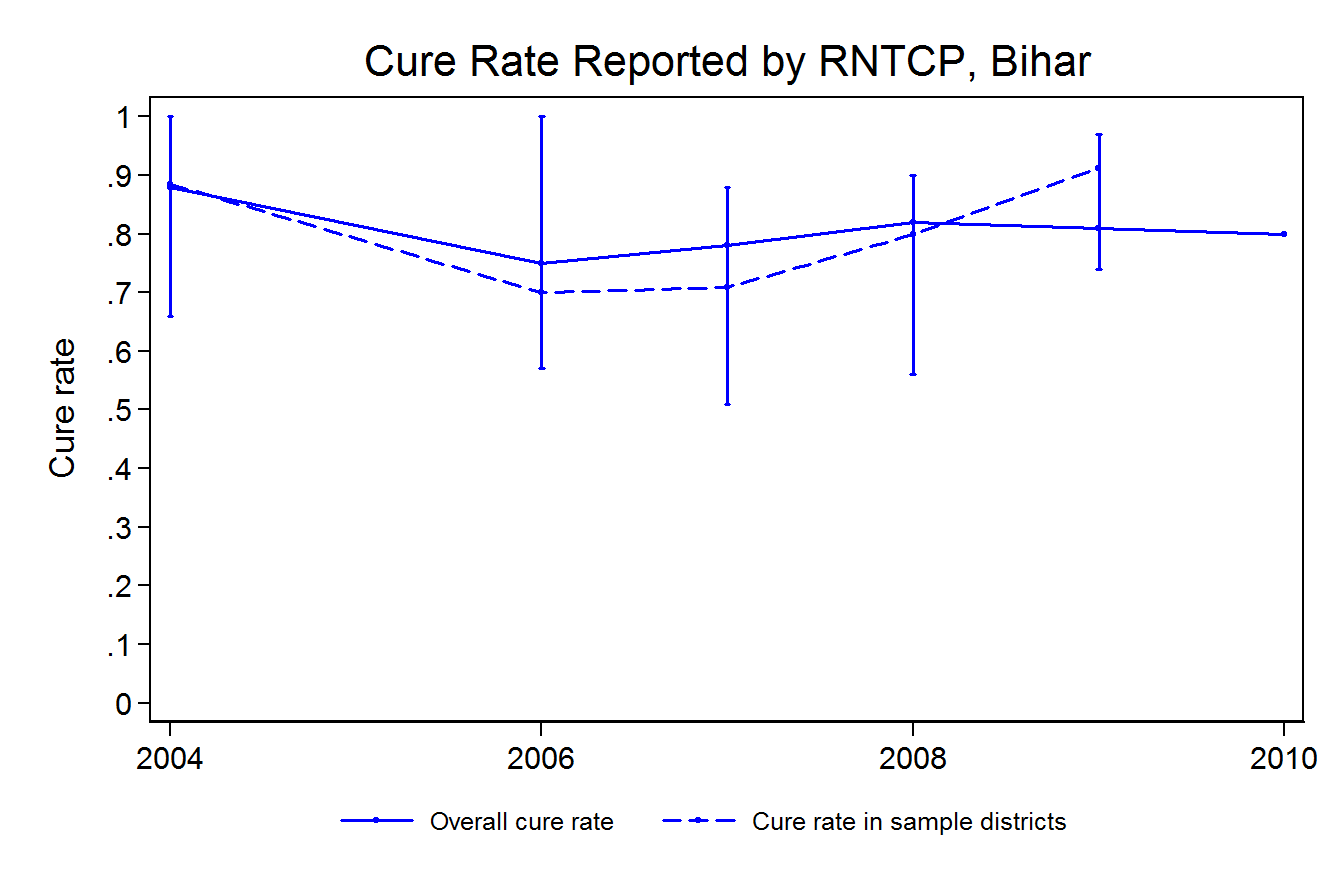

Supplement: Additional file 10 — Cure Rate Reported by RNTCP. [file 1471-2458-14-418-S10.docx]

**Additional file 11: Default Rates Reported by RNTCP, Bihar**


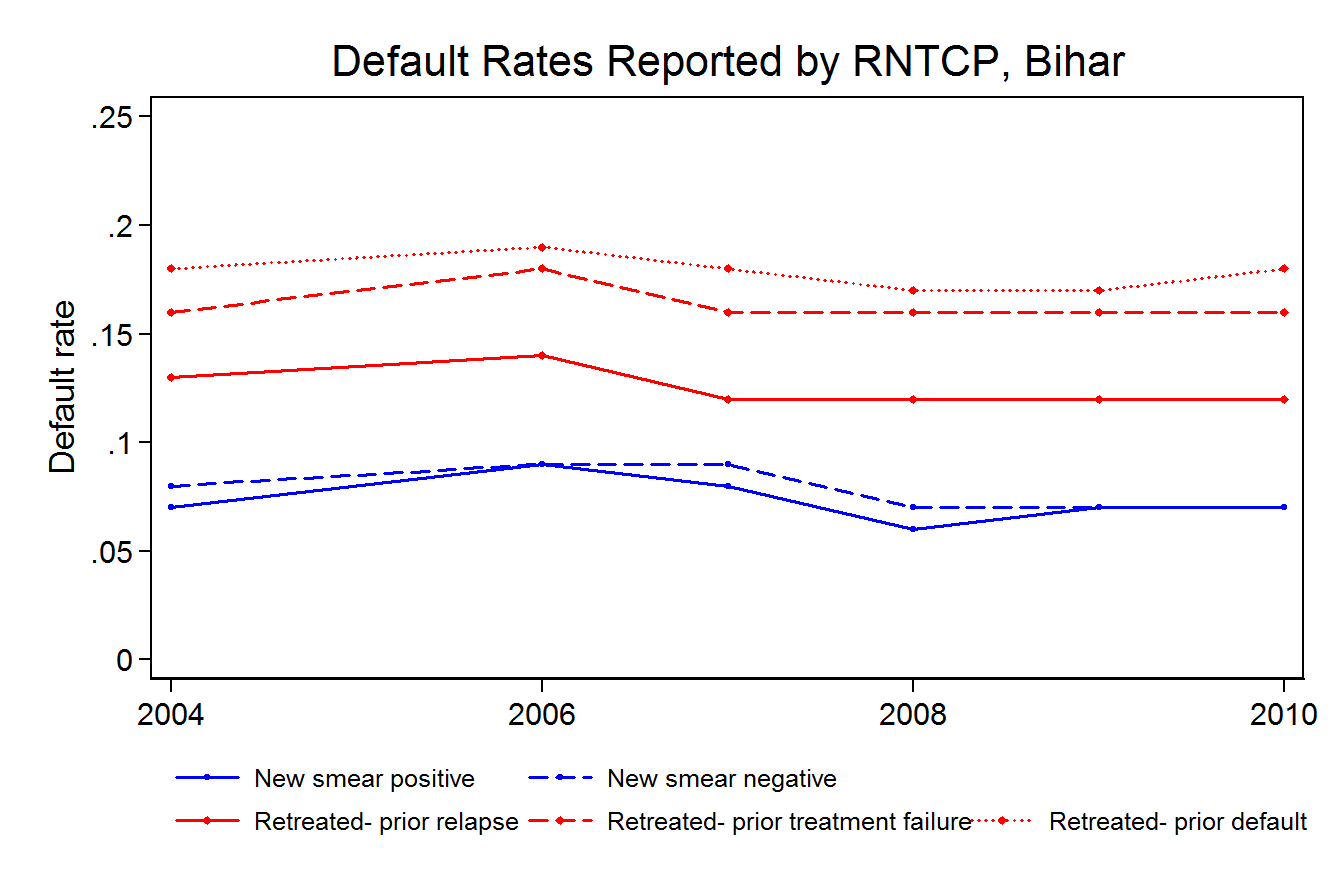

Supplement: Additional file 11 — Default Rates Reported by RNTCP, Bihar. [file 1471-2458-14-418-S11.docx]
