# Supplementary material for: Tuberculosis treatment discontinuation and symptom persistence: an observational study of Bihar, India’s public care system covering >100,000,000 inhabitants
Source: BMC Public Health. 2014 May 1;14:418. doi: 10.1186/1471-2458-14-418 (PMC4041057; doi:10.1186/1471-2458-14-418)
Supplement: Additional file 12: Table S7 — Treatment Default Prior to 8 Months Among Previously Treated Patients. [file 1471-2458-14-418-S12.docx]

**Additional file 12: Table S7: Treatment Default Prior to 8 Months Among Previously Treated Patients**

|  | **Univariate Regression** | | **Multivariate Regressions** | | | |
| --- | --- | --- | --- | --- | --- | --- |
|  | **Default Prior to 8 Months** | | **Default Prior to 8 Months** | | **Default Prior to 6 Months** | |
|  | **OR** | **(95% CI)** | **OR** | **(95% CI)** | **OR** | **(95% CI)** |
|  |  |  |  |  |  |  |
| **Prior TB Status** |  |  |  |  |  |  |
| **Prior TB & Completed Prior Treatment** | 0.24 | (0.04 - 1.66) | 0.24 | (0.01 - 4.19) | 0.15* | (0.03 - 0.73) |
|  |  |  |  |  |  |  |
| **Current Illness Treatment and Illness Characteristics** |  |  |  |  |  |  |
| **Total Weeks from Symptom Onset**  **to Treatment Initiation** | 1.05 | (0.91 - 1.22) | 0.98 | (0.82 - 1.18) | 1.04 | (0.90 - 1.20) |
| **Number of Providers Visited** | 3.22 | (0.24 - 43.87) | 1.24 | (0.09 - 16.83) | 3.08 | (0.46 - 20.45) |
| **Treatment or Medication Fees** | 1.80 | (0.13 - 25.39) | 19.71 | (0.41 - 952.49) | 1.13 | (0.08 - 16.48) |
| **Travel Costs** | 0.22 | (0.01 - 7.31) | 0.07 | (0.00 - 5.94) | 1.43 | (0.13 - 16.08) |
| **Treatment, Medication and Travel Costs** | 1.19 | (0.03 - 50.33) | 0.58 | (0.00 - 144.20) | 1.04 | (0.05 - 21.04) |
| **2 or Fewer Symptoms at Treatment Initiation**** | 2.72 | (0.20 - 36.87) | 6.56 | (0.72 - 60.04) | 4.20* | (1.26 - 13.92) |
| **3-4 Symptoms at Treatment Initiation**** | 1.49 | (0.42 - 5.25) | 1.42 | (0.20 - 9.97) | 2.55 | (0.66 - 9.95) |
|  |  |  |  |  |  |  |
| **Patient and Household Characteristics** |  |  |  |  |  |  |
| **Male** | 0.86 | (0.21 - 3.42) | 1.36 | (0.41 - 4.48) | 1.02 | (0.39 - 2.69) |
| **Age** | 1.05 | (0.90 - 1.22) | 1.10 | (0.89 - 1.36) | 0.84 | (0.70 - 1.01) |
| **Age Squared** | 1.00 | (1.00 - 1.00) | 1.00 | (1.00 - 1.00) | 1.00* | (1.00 - 1.00) |
| **Education** | 0.95 | (0.85 - 1.06) | 0.96 | (0.77 - 1.21) | 0.75* | (0.58 - 0.98) |
| **Hindu** | 0.31 | (0.02 - 4.50) | 0.32 | (0.02 - 5.04) | 0.25* | (0.08 - 0.78) |
| **Scheduled Caste, Tribe, Other Backwards Class** | 0.16 | (0.02 - 1.51) | 0.13 | (0.01 - 1.27) | 0.36 | (0.08 - 1.71) |
| **Number of Kids** | 1.38 | (0.98 - 1.96) | 1.41 | (0.84 - 2.37) | 0.80 | (0.53 - 1.20) |
| **Household Size** | 1.03 | (0.81 - 1.32) | 0.94 | (0.66 - 1.34) | 1.35 | (0.97 - 1.88) |
| **Poor** | 2.05 | (0.72 - 5.90) | 3.54 | (0.90 - 13.95) | 0.76 | (0.14 - 4.11) |
| **Middle Income** | 4.55* | (1.09 - 19.04) | 6.09* | (1.00 - 37.10) | 0.99 | (0.30 - 3.20) |
|  |  |  |  |  |  |  |
| **Observations** | 151 | | 151 | | 196 | |

* p<0.05

** Comparator group is ≥5 Symptoms at Treatment Initiation
